# New Allosteric Modulators of AMPA Receptors: Synthesis and Study of Their Functional Activity by Radioligand-Receptor Binding Analysis

**DOI:** 10.3390/ijms241210293

**Published:** 2023-06-18

**Authors:** Elena A. Golubeva, Mstislav I. Lavrov, Polina N. Veremeeva, Tatiana V. Vyunova, Konstantin V. Shevchenko, Maxim A. Topchiy, Andrey F. Asachenko, Vladimir A. Palyulin

**Affiliations:** 1 Department of Chemistry, Lomonosov Moscow State University, 119991 Moscow, Russia; 2 Laboratory of Molecular Pharmacology of Peptides, Institute of Molecular Genetics, National Research Centre Kurchatov Institute, 123182 Moscow, Russia; 3 A.V. Topchiev Institute of Petrochemical Synthesis, Russian Academy of Sciences, 119991 Moscow, Russia

**Keywords:** AMPA receptor allosteric modulators, CNS pathologies, radioligand binding, [^3^H]PAM-43, cross-coupling reaction, heterogeneous hydrogenation

## Abstract

The synthetic approaches to three new AMPA receptor modulators—derivatives of 1,11-dimethyl-3,6,9-triazatricyclo[7.3.1.1^3,11^]tetradecane-4,8,12-trione—had been developed and all steps of synthesis were optimized. The structures of the compounds contain tricyclic cage and indane fragments necessary for binding with the target receptor. Their physiological activity was studied by radioligand-receptor binding analysis using [^3^H]PAM-43 as a reference ligand, which is a highly potent positive allosteric modulator of AMPA receptors. The results of radioligand-binding studies indicated the high potency of two synthesized compounds to bind with the same targets as positive allosteric modulator PAM-43 (at least on AMPA receptors). We suggest that the Glu-dependent specific binding site of [^3^H]PAM-43 or the receptor containing this site may be one of the targets of the new compounds. We also suggest that enhanced radioligand binding may indicate the existence of synergistic effects of compounds **11b** and **11c** with respect to PAM-43 binding to the targets. At the same time, these compounds may not compete directly with PAM-43 for its specific binding sites but bind to other specific sites of this biotarget, changing its conformation and thereby causing a synergistic effect of cooperative interaction. It can be expected that the newly synthesized compounds will also have pronounced effects on the glutamatergic system of the mammalian brain.

## 1. Introduction

Synaptic transmission is the main pathway of neural communication, which forms the basis for the analysis, systematization, and memorization of information in the central nervous system (CNS) [1,2,3]. Rapid excitatory synaptic transmission in the CNS is mainly mediated via the AMPA subtype of glutamate receptors [4,5], and that makes these receptors key targets for the regulation of synaptic excitation.

When AMPA receptors are activated by glutamate, they rapidly desensitize [6,7,8,9]. To date, a number of allosteric modulators of these receptors are known, which by themselves do not activate AMPA receptors but increase the current induced by the agonist, thus slowing down the rate of desensitization [10,11,12,13]. This fact largely determines a wide range of possible therapeutic applications of such modulators. One of the significant neurophysiological actions of allosteric modulators of AMPA receptors is synaptic plasticity [14,15,16], playing a key role in the mechanism of neural memory. The important therapeutic potential of AMPA receptor allosteric modulators is also based on their ability to significantly increase the expression of neurotrophic factors, namely, nerve growth factor (NGF) and brain-derived neurotrophic factor (BDNF), which in turn lead to the regeneration of nervous tissue and protection of neurons from neurotoxic damage [17,18,19,20,21].

Thus, the above facts make AMPA receptor allosteric modulators promising candidates for the development of drugs for the treatment of cognitive disorders (including early stages of Alzheimer’s disease), depression, and a number of other CNS pathologies [4,5], attention-deficit/hyperactivity disorder (ADHD) and mood disorders [22,23], as well as drug-induced respiratory depression [24]. The key advantage of allosteric modulators of AMPA receptors is that (unlike agonists or antagonists) they potentiate the receptor only in the presence of an endogenous ligand and, therefore, cause fewer side effects [25,26].

However, despite the encouraging preclinical results, most AMPA receptor modulators to date have not progressed beyond early clinical development [27]. These problems are associated with insufficiently relevant in vitro and in vivo models of neurological and neuropsychiatric disorders, issues of pharmacodynamics and pharmacokinetics, as well as with a limited understanding of the processes underlying the activity and specificity of the action of modulators: despite significant progress in the development of cell models [28] and animal models [29,30,31,32] of neurodegenerative diseases, none of them is yet able to fully capture all the factors associated with these complex pathologies. Nevertheless, the development of new chemotypes of AMPA receptor modulators has become a priority for many pharmaceutical companies [27].

Earlier extensive research in the field of molecular modeling and molecular dynamics studies of complexes of various ligands with AMPA receptors was carried out, and it was experimentally shown that some bispidine derivatives are highly active with respect to the glutamatergic system [33,34]. As a result of these studies, new potential allosteric modulators based on a bispidine moiety-containing tricyclic scaffold were developed. In particular, the derivatives of 1,11-dimethyl-3,6,9-triazatricyclo[7.3.1.1^3,11^]tetradecane-4,8,12-trione in electrophysiological studies using the patch clamp technique, showed pronounced modulatory effects (both positive and negative) in subnanomolar concentrations with respect to AMPA receptors [35,36,37,38,39]. An important advantage of both positive and negative allosteric modulators of AMPA receptors is that they suppress the process of desensitization—the inability of the receptor to re-transit into an activated state, despite the presence of an agonist.

Nowadays, the radioligand-receptor binding analysis using tritium-labeled molecules is widely and successfully applied to study the molecular mechanisms of action of biologically active compounds and is one of the best, fast, and most reliable techniques [40]. This approach makes it possible to identify molecular targets—specific receptors through which the substance affects cell metabolism—and to characterize the interactions of ligands with detected receptors qualitatively and quantitatively. It also allows one to conduct qualitative and quantitative studies of the spectrum of interactions of the developed new drugs with membrane receptors of various types [41], to compile a receptor profile of a biologically active compound [42], to study the relationships between the structure of ligands and their action [43], to conduct a comparative assessment of compounds by their interaction with the biological targets [44,45], and to analyze pharmacokinetics [46].

In this article, we have developed and optimized the synthetic approaches to several derivatives of 1,11-dimethyl-3,6,9-triazatricyclo[7.3.1.1^3,11^]tetradecane-4,8,12-trione as new potential AMPA receptor modulators and studied their functional activity by radioligand-receptor binding analysis using tritiated compound PAM-43 (an active, positive AMPA receptor modulator) as a reference ligand.

## 2. Results and Discussion

### 2.1. Synthesis of Target Compounds ***11a**–**c*** 

**Scheme 1:**
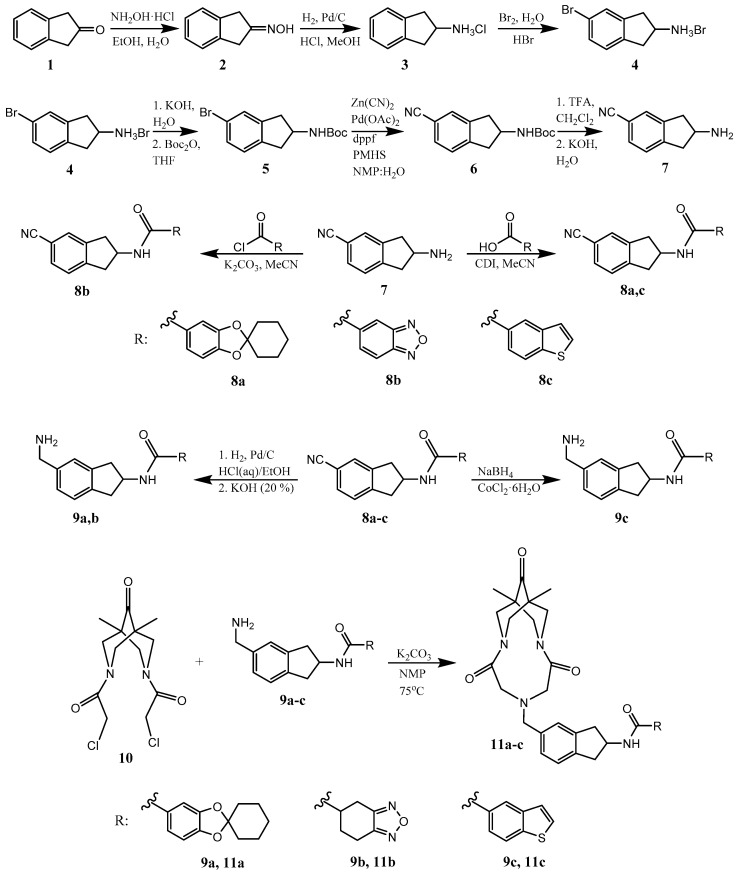
Synthesis of target compounds **11a**–**c**.

The key tricyclic compounds **11a**–**c** were synthesized from indan-2-one **1** according to Scheme 1. Oxime **2**, obtained from indan-2-one **1**, was reduced to amine **3** hydrochloride. Then, bromo-derivative **4** was synthesized by electrophilic aromatic bromination. It underwent Pd-catalyzed cyanation after preliminary protection of the NH_2_-group. Further, deprotection gave the key intermediate **7** for obtaining a series of amides **8a**–**c**. The reduction led to amines **9a**–**c**, which were alkylated with dichloro derivative **10** to provide corresponding tricyclic compounds **11a**–**c**.

For the obtaining of indan-2-one oxime **2**, a well-known method [47,48] was modified. The synthesis was carried out by mixing a solution of indan-2-one **1** and hydroxylamine hydrochloride in the EtOH/H_2_O system (1:1). The proposed changes make it possible to avoid the addition of pyridine and thus facilitate the isolation of the product. For synthesis of indan-2-ylamine hydrochloride **3** a widely used method based on heterogeneous hydrogenation [47,48] was modified. It was shown that the addition of PdCl_2_ was not required for the reaction. Conducting the reaction at a hydrogen pressure of 5 atm (in an autoclave) increases the yield, reduces the reaction time, and prevents the formation of byproducts. That leads to a clean product without the need for additional purification, thus making it possible to obtain indan-2-ylamine hydrochloride **3** with the yield of 91%.

Selective bromination of indan-2-ylamine hydrochloride **3** presents certain difficulties since the rates of monobromination reaction and dibromination reaction in an aqueous solution are similar. As a result of the procedure optimization, it was found that the key factor was the amount of water. The optimal H_2_O/indan-2-ylamine hydrochloride ratio of 4.5 mL/g allows the reaction to be carried out selectively in good yield due to the fact that the monobromo derivative formed in the course of the reaction precipitates and does not undergo the second bromination.

The cyano-derivative **6** was obtained from the Boc-protected compound **5** by a cross-coupling reaction. First, the reaction with CuCN was conducted under various conditions, but the yields were low. Then, palladium catalysts were tested. As a result of the procedure optimization, it was found that the key factor was the addition of the reducing agent PMHS (polymethylhydrosiloxane), which significantly increased the yield. The best results were achieved using dppf (1,1′-bis(diphenylphosphino)ferrocene) as a ligand, Zn(CN)_2_ as a source of CN^–^ and PMHS. Further, deprotection provided the key intermediate **7** for obtaining a series of amides **8a**–**c**. Despite two additional stages of protection and deprotection, this way is preferable in comparison with acylation–cyanation, since it allows branching of the synthetic scheme at a later stage and does not require the cyanation technique optimization for each amide.

Compounds **8a**,**c** were synthesized by acylation of amine **7** with commercially available acids in the presence of CDI. It should be noted that an alternative method was used for compound **8b** using the corresponding acyl chloride, since the reaction with the acid and CDI did not proceed.

Amines **9a**–**c** were obtained by the reduction of corresponding nitriles **8a**–**c**. The key problem of the CN group reduction is to ensure selectivity in the presence of an amide group. That makes it impossible to use the classical methods of reduction by complex hydrides.

Heterogeneous hydrogenation of the corresponding nitriles was carried out in an autoclave using 10% Pd/C as a catalyst. By varying the reaction conditions (time, pressure, and concentration of HCl), it was found that the key factor for the reaction is the addition of 1.5 eq. concentrated HCl. The developed method has been successfully applied to the synthesis of amines **9a**,**b** in the form of hydrochlorides with high yields. It should be noted that in the case of compound **8b**, such conditions result in the reduction in both the CN group and benzene ring [49]; therefore, nitrile **8b** was used for the synthesis of amine **9b** with the alicyclic ring. Amine **9c** could not be obtained by heterogeneous hydrogenation. Therefore, the method of nitrile reduction using NaBH_4_ and CoCl_2_·6H_2_O was used [50,51,52]. This method has been modified for amine **9c**. The main factor is the reduction time. With a short reaction time, the reduction did not proceed completely, and the product was isolated as a mixture with the initial nitrile **8c**. If the reaction is carried out for too long, side processes occur that significantly reduce the yield of the final compound. The best results were achieved when the reaction was carried out for 4 h.

The key tricyclic compounds **11a**–**c** were synthesized by double alkylation of the corresponding amines **9a**–**c** with dichloro derivative **10**. Compound **10** was obtained using a modified method for the acylation of 5,7-dimethyl-1,3-diazaadamantan-6-one in a two-phase CHCl_3_/H_2_O system, which makes it easier to isolate the product and increases the yield [36].

The reaction of alkylation was optimized by varying the base, solvent, and temperature. K_2_CO_3_ and Cs_2_CO_3_ as bases gave the best results; finally, K_2_CO_3_ was used in the reaction. NMP (N-methyl-2-pyrrolidone) was used as a solvent because it completely dissolves the starting amines and has a rather high boiling point to keep the necessary reaction temperature.

### 2.2. Biological Studies

**Figure 1:**
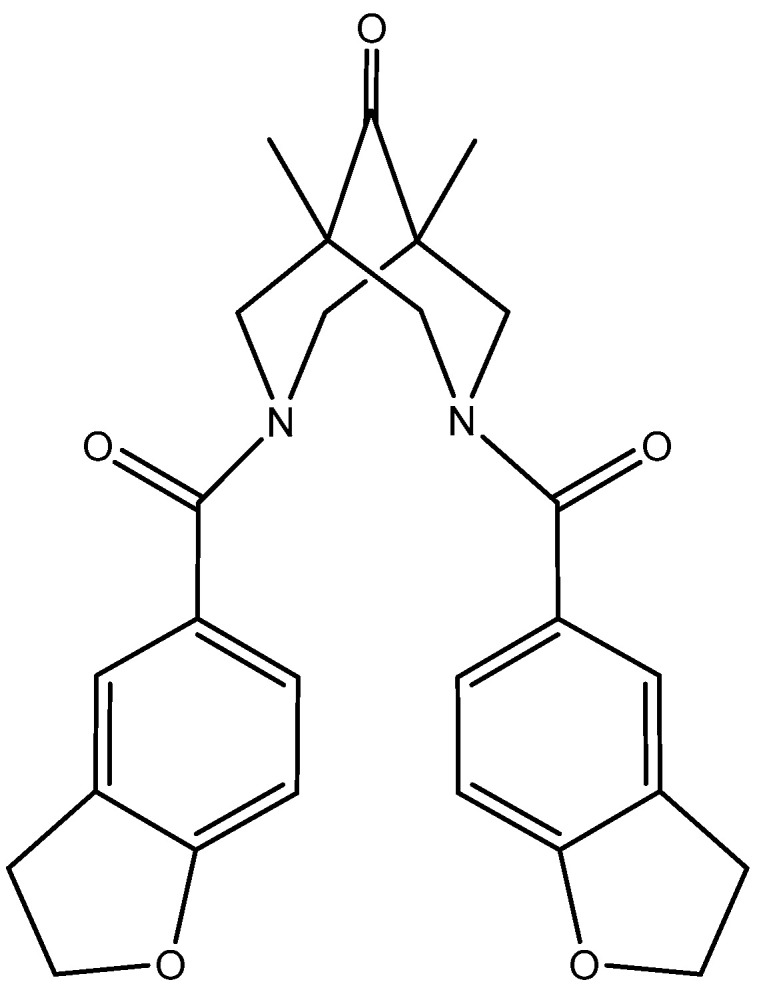
PAM-43 structure.

To make a conclusion about the biological activity of synthesized potential AMPA receptor modulators, we performed a selective functional screening of the ligand-receptor binding. At this stage, we tried to avoid commonly used AMPA-receptor-specific radioligands, such as [^3^H]AMPA, [^3^H]fluorowillardiine, or similar, which have many different receptor targets as a rule, and many contradictory effects have been observed for various allosteric AMPA receptor modulators when these kinds of radioligands were used (see, e.g., [53,54,55]). That is why we have chosen the ligand whose targets are most likely to coincide (or overlap most tightly) with the targets of the synthesized compounds. The tritium-labeled compound PAM-43 ([^3^H]PAM-43 [56]) was used as a basic ligand. PAM-43 (a derivative of 3,7-diazabicyclo[3.3.1]nonane [57], Figure 1) is a positive AMPA receptor modulator, a proposed new drug candidate that has a stimulating effect on memory, learning, and integrative brain functions. PAM-43 (in a dose range of 0.05–0.5 mg/kg) stimulates memory and cognitive abilities in rats [58]. The chemical structure of PAM-43 is similar to that of some previously known synthetic allosteric modulators of AMPA receptors, and the molecular modeling also confirmed the ability of PAM-43 to act as an allosteric modulator on AMPA receptors [59]. It has also been shown that PAM-43 (as an allosteric modulator) is able to potentiate ionotropic GluR currents in rat cerebellar Purkinje neurons in a concentration-dependent manner [57]. The radioligand-receptor binding studies revealed the existence of at least two different sites of [^3^H]PAM-43 specific bindings localized on rat brain cells’ plasmatic membranes. These sites are different: one of them is Glu-independent and characterized by a lower affinity, and the other has a higher affinity but appears only in the presence of free Glu [57]. Theoretically, the molecular targets of PAM-43 and synthesized novel potential AMPA receptor modulators are tightly close or overlap. Thus, we assume that the existence of noticed effects of low concentrations of synthesized modulators on [^3^H]PAM-43 specific bindings will likely indicate the presence of functional and biological activities in tested substances.

**Figure 2:**
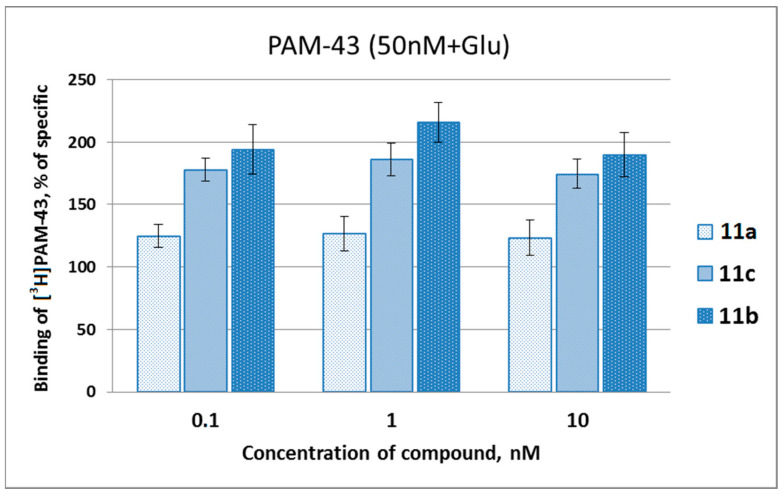
Specific binding of [^3^H]PAM-43 (50 nM, L-Glu saturation) in the presence of different concentrations of synthesized compounds to corresponding receptors on rat cerebral cortex neurons’ plasmatic membranes. The abscissa represents the concentration of a compound in the incubation mixture, nmol per liter. The ordinate represents the proportion of [^3^H]PAM-43 (50 nM) specific binding, %. A value of 100% corresponds to [^3^H]PAM-43 (50 nM) specific binding determined in the presence of unlabeled PAM-43 (250 μM). L-Glu (500 μM) was preliminarily added to plasmatic membranes 30 min before the incubation with radioligand and compounds; the final concentration of L-Glu in the binding assay was 250 μM.

**Figure 3:**
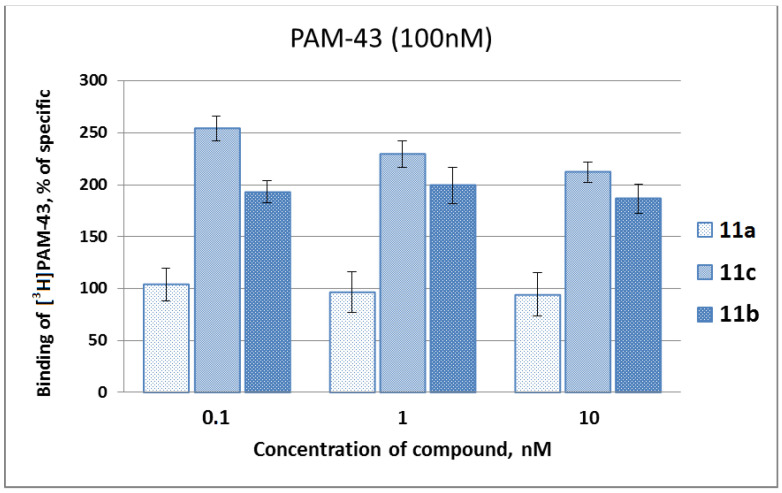
Specific binding of [^3^H]PAM-43 (100 nM) in the presence of different concentrations of synthesized compounds to corresponding receptors on rat cerebral cortex neurons’ plasmatic membranes. The abscissa represents the concentration of a compound in the incubation mixture, nmol per liter. The ordinate represents the proportion of [^3^H]PAM-43 (100 nM) specific binding, %. A value of 100% corresponds to [^3^H]PAM-43 (100 nM) specific binding determined in the presence of unlabeled PAM-43 (250 μM).

The effect of compounds **11a**–**c** on specific binding of [^3^H]PAM-43 was tested for both its sites—high and low affine (Figure 2 and Figure 3). All the compounds potentiated the binding of [^3^H]PAM-43 on its high-affinity Glu-dependent site, but with different efficiency (Figure 2). Compound **11b** was the most active and increased the binding almost twice in the concentration range of 0.1–10 nM. Compound **11c** showed less efficacy, and compound **11a** showed no more than a 20% increase in [^3^H]PAM-43 binding in all the tested concentrations of the compound.

So, the data obtained show that the Glu-dependent site of [^3^H]PAM-43 specific bindings, or a receptor containing this site, may be one of the targets of compounds **11b** and **11c**. We also assume that the increase in binding of the radioligand may be related to the effects of cooperativity of specific binding. The compounds **11b** and **11c** may bind first but facilitate subsequent binding of PAM-43 or increase its retention time on the receptor. In this light, we may expect the same or higher functional and, probably, the biological activity of compounds **11b** and **11c**.

The second site of [^3^H]PAM-43 specific binding is Glu-independent but possesses low affinity. Compounds **11b** and **11c** also had a positive effect on this site—they doubled the binding (Figure 3). The compound **11a** was inactive, but we cannot exclude its efficacy at some higher concentrations.

So, both sites of [^3^H]PAM-43 specific bindings showed high sensitivity to low concentrations of compounds **11b** and **11c**, and the effect in both cases was twofold. Both compounds have demonstrated a significant increase in PAM-43 binding. According to the available data, AMPA receptors have multiple binding sites [60], so we can suppose that the binding sites of compounds **11b** and **11c** in the receptors at least partially do not coincide with the binding sites of PAM-43, however binding of these compounds leads to a pronounced synergistic increase in PAM-43 binding. That confirms that biotargets of new compounds and biotargets of PAM-43 are interrelated. Thus, we can expect the biological effects of new compounds to be similar to those of PAM-43. The compound **11a** almost had no effects, but we cannot exclude that **11a** may target the same as PAM-43 sites but with lower affinity. This is a matter of separate follow-up studies.

## 3. Materials and Methods

### 3.1. Chemistry

*General information:* The chemicals (indan-2-one, benzo[c][1,2,5]oxadiazole-5-carboxylic acid, carbonyldiimidazole,) were purchased from the commercial sources (Sigma-Aldrich, St. Louis, MO, USA) and used as received. 3,7-bis(chloroacetyl)-1,5-dimethyl-3,7-diazabicyclo[3.3.1]nonan-9-one were prepared according to the published methods [36] and characterized by NMR spectra. NMR spectra were recorded on the Agilent 400-MR (Bruker, Karlsruhe, Germany) spectrometer (400.0 MHz for ^1^H; 100.6 MHz for ^13^C) at room temperature; chemical shifts (δ) were measured with reference to the solvents: CDCl_3_ for ^1^H (δ = 7.26 ppm), ^13^C (δ = 77.16 ppm), DMSO-d6 for ^1^H (δ = 2.50 ppm), ^13^C (δ = 39.50 ppm) and CD_3_OD for ^1^H (δ = 3.31 ppm), ^13^C (δ = 49.00 ppm). Chemical shifts (δ) are given in ppm; J values are given in Hz. Accurate mass measurements (HRMS) were performed on a Bruker micrOTOF II instrument (Bruker, Karlsruhe, Germany) using electrospray ionization (ESI). The measurements were done in a positive ion mode (interface capillary voltage 4500 V). Analytical thin layer chromatography was carried out with Merck Silica Gel 60 plates (supported on aluminum, Sigma-Aldrich, St. Louis, MO, USA); the detection was done by UV lamp (254 and 365 nm) and chemical staining (solution of ninhydrin in EtOH). Column chromatography was performed on Merck Silica Gel 60 (Sigma-Aldrich, St. Louis, MO, USA). Original ^1^H and ^13^C NMR spectra for compounds **8a**–**c**, **9a**–**c**, and **11a**–**c** are given in Supplementary Materials.

*Indan-2-one oxime (***2***).* A mixture of indan-2-one **1** (26.42 g, 0.200 mol) and NH_2_OH·HCl (17.32 g, 0.250 mol) in EtOH/H_2_O (1:1, 1000 mL) was stirred first for 1.5 h, then under heating at 50 °C for additional 1 h. The reaction mixture was cooled to room temperature. The precipitate formed was filtered off and washed with distilled water. The product was recrystallized from EtOH (150 mL). This yielded 20.89 g (0.141 mol, 71%) of pure indan-2-one oxime **2** as needle crystals. mp: 150–153 °C [47], spectral data [61].

*Indan-2-ylamine hydrochloride (***3***).* A mixture of indan-2-one oxime **2** (5.40 g, 0.037 mol) and 10% Pd/C (3.62 g, 9 mol. %) in 1M HCl/MeOH_abs_ (110 mL) was placed into a glass autoclave, and hydrogen was passed through (p = 5 atm) while stirring for 6 h. The reaction mixture was filtered through celite, washed with MeOH, and the solvent was distilled off. This yielded 5.64 g (0.033 mol, 91%) of pure indan-2-ylamine hydrochloride **3** as a white crystalline product. mp: 244–245 °C [47], spectral data [62].

*5-Bromoindan-2-ylamine hydrobromide (***4***).* To a solution of indan-2-ylamine hydrochloride **3** (2.82 g, 16.6 mmol) in distilled water (12.6 mL), bromine (940 μL, 18.3 mmol) was added dropwise while stirring. The reaction mixture was stirred for 9 h. After that, HBr_conc_ (ω = 48%, 2.82 mL, 24.9 mmol) was added, and the reaction mixture was stirred for additional 3 h. The precipitate formed was filtered off and washed with distilled water (4.5 mL). The product was dissolved in EtOH (37 mL), precipitated with Et_2_O and filtered off. This yielded 3.15 g (13.3 mmol, 65%) of pure 5-bromoindan-2-ylamine hydrobromide **4** as a crystalline product. mp: >300 °C [63], spectral data [63].

*Tert-butyl(5-bromoindan-2-yl)carbamate (***5***).* A 20% aqueous solution of KOH was added to a suspension of 5-bromoindan-2-ylamine hydrobromide **4** (3.11 g, 10.6 mmol) in distilled water (pH 10). The resulting mixture was extracted with CH_2_Cl_2_, the combined organic phases were dried over anhydrous Na_2_SO_4_, filtered, and then the solvent was distilled off. The product was dissolved in absolute tetrahydrofuran (40 mL), then Boc_2_O (2.43 g, 11.1 mmol) was added, and the reaction mixture was stirred for 1.5 h. After that, the solvent was distilled off. The resulting product was treated with n-hexane (9 mL) and filtered off. This yielded 3.27 g (10.5 mmol, 99%) of pure tert-butyl(5-bromoindan-2-yl)carbamate **5** as a white solid. mp: 167–169 °C, spectral data [64].

*Tert-butyl(5-cyanoindan-2-yl)carbamate (***6***).* A mixture of tert-butyl(5-bromoindan-2-yl)carbamate **5** (936 mg, 3 mmol), Pd(OAc)_2_ (13.5 mg, 0.06 mmol, 2 mol. %), and dppf (33.3 mg, 0.06 mmol, 2 mol. %) in N-methyl-2-pyrrolidone/H_2_O (10:1 by volume, 3.3 mL) was stirred for 1 min. Then, PMHS (100 mg) and Zn(CN)_2_ (180 mg, 1.53 mmol) were added, and the reaction mixture was stirred under heating at 120 °C for 12 h. Afterward, the reaction mixture was cooled to room temperature, and the solvent was distilled off. The resulting product was purified by flash chromatography (eluents CH_2_Cl_2_, CH_2_Cl_2_/light petroleum ether (1:1)). This yielded 541 mg (2.1 mmol, 70%) of pure tert-butyl(5-cyanoindan-2-yl)carbamate **6** as a white solid. mp: 161–162 °C, spectral data [64].

*2-Aminoindan-5-carbonitrile (**7**).* To a solution of tert-butyl(5-cyanoindan-2-yl)carbamate **6** (980 mg, 3.79 mmol) in CH_2_Cl_2_ (25 mL), trifluoroacetic acid (2.82 mL, 36.9 mmol) was added, and the reaction mixture was stirred for 2 h. Afterward, distilled water (10 mL) and a 20% aqueous solution of KOH were added to the reaction mixture (pH = 10). The organic phase was separated; the aqueous phase was extracted with CH_2_Cl_2_. The combined organic phases were dried over anhydrous Na_2_SO_4_, then filtered, and the solvent was distilled off. This yielded 594 mg (3.75 mmol, 99%) of pure 2-aminoindan-5-carbonitrile **7** as a colorless oily product. Spectral data (in the form of hydrochloride) [64].

*N-(5-Cyanoindan-2-yl)benzo[c]*[1,2,5]*oxadiazole-5-carboxamide (**8*****b***).* To a solution of benzo[c][1,2,5]oxadiazole-5-carboxylic acid chloride (242 mg, 1.33 mmol) in absolute acetonitrile, K_2_CO_3_ (350 mg, 2.53 mmol) and solution of 2-aminoindan-5-carbonitrile **7** (200 mg, 1.26 mmol) in absolute acetonitrile were added. The resulting mixture was stirred under heating at 40 °C until complete consumption of the starting amine (TLC control R_f_ = 0.07 in CHCl_3_/EtOH (20:1) system). Afterward, the reaction mixture was cooled to room temperature and filtered. The solid residue was washed with absolute acetonitrile until no more product was washed out (TLC control). The filtrates were combined, and the solvent was distilled off. This yielded pure N-(5-cyanoindan-2-yl)benzo[c][1,2,5]oxadiazole-5-carboxamide **8b** (95%) as a light brown crystalline product. R_f_ = 0.69 (CHCl_3_/EtOH (20:1)). mp: 187–189 °C. ^1^H NMR (DMSO-d6), δ: 3.00–3.09 (m, 2H), 3.31–3.40 (m, 2H), 4.74 (m, 1H), 7.46 (d, 1H, ^3^J 7.8 Hz), 7.62 (dd, 1H, ^3^J 7.8 Hz, ^4^J 1.4 Hz), 7.71 (s, 1H), 7.94 (dd, 1H, ^3^J 9.4 Hz, ^4^J 1.3 Hz), 8.11 (dd, 1H, ^3^J 9.4 Hz, ^5^J 1.0 Hz), 8.52 (t, 1H, J 1.2 Hz), 9.10 (d, 1H, ^3^J 6.7 Hz). ^13^C NMR (DMSO-d6), δ: 38.58, 39.12, 50.68, 109.18, 115.81, 116.28, 119.34, 125.74, 128.17, 130.76, 131.56, 137.81, 142.94, 147.66, 148.81, 149.09, 164.55. HRMS (ESI), *m*/*z* 305.1032 (calc. C_17_H_12_N_4_O_2_ [M + H]^+^, *m*/*z*: 305.1033).

*General procedure for acylation of 2-aminoindan-5-carbonitrile***7**. A mixture of corresponding acid (1 eq.) and carbonyldiimidazole (1.05 eq.) in absolute acetonitrile was stirred until the formation of corresponding imidazolide (TLC control). Then, a solution of 2-aminoindan-5-carbonitrile **7** (1 eq.) in absolute acetonitrile was added, and the reaction mixture was stirred until complete consumption of the starting amine (TLC control R_f_ = 0.07 in CHCl_3_/EtOH (20:1) system). After that, the solvent was distilled off.

*N-(5-Cyanoindan-2-yl)spiro[1,3-benzodioxole-2,1′-cyclohexane]-5-carboxamide (***8a***).* The resulting product was treated with Et_2_O and filtered off. This yielded pure N-(5-cyanoindan-2-yl)spiro[1,3-benzodioxole-2,1′-cyclohexane]-5-carboxamide **8a** (81%) as a light brown solid. R_f_ = 0.79 (CHCl_3_/EtOH (20:1)). mp: 149–150 °C. ^1^H NMR (CDCl_3_), δ: 1.50 (m, 2H), 1.72 (m, 4H), 1.89 (dist. t, 4H), 2.93–3.00 (m, 2H), 3.40–3.48 (m, 2H), 4.93 (m, 1H), 6.17 (d, 1H, ^3^J 7.3 Hz), 6.71 (d, 1H, ^3^J 8.1 Hz), 7.14 (d, 1H, ^4^J 1.8 Hz), 7.20 (dd, 1H, ^3^J 8.1 Hz, ^4^J 1.8 Hz), 7.34 (d, 1H, ^3^J 7.8 Hz), 7.50 (br. d, 1H, ^3^J 7.8 Hz), 7.53 (s, 1H). ^13^C NMR (CDCl_3_), δ: 22.66, 23.99, 34.74, 39.24, 39.91, 50.51, 106.88, 107.36, 110.13, 118.80, 119.62, 120.60, 125.22, 127.08, 127.91, 130.63, 141.91, 146.46, 147.43, 150.05, 166.65. HRMS (ESI), *m*/*z* 375.1699 (calc. C_23_H_22_N_2_O_3_ [M + H]^+^, *m*/*z*: 375.1703).

*N-(5-Cyanoindan-2-yl)benzo[b]thiophene-5-carboxamide (***8c***).* The resulting product was recrystallized from acetonitrile. This yielded pure N-(5-cyanoindan-2-yl)benzo[b]thiophene-5-carboxamide **8c** (85%) as a white solid. R_f_ = 0.78 (CHCl_3_/EtOH (20:1)). mp: 185–187 °C. ^1^H NMR (CDCl_3_), δ: 2.99–3.07 (m, 2H), 3.43–3.51 (m, 2H), 5.00 (m, 1H), 6.50 (d, 1H, ^3^J 6.6 Hz), 7.34–7.38 (m, 2H), 7.49–7.53 (m, 3H), 7.69 (dd, 1H, ^3^J 8.4 Hz, ^4^J 1.3 Hz), 7.90 (d, 1H, ^3^J 8.4 Hz), 8.22 (d, 1H, ^4^J 1.0 Hz). ^13^C NMR (CDCl_3_), δ: 39.20, 39.88, 50.59, 110.13, 118.81, 122.04, 122.17, 122.20, 123.74, 125.21, 127.59, 127.90, 130.06, 130.63, 138.98, 141.88, 142.37, 146.42, 167.32. HRMS (ESI), *m*/*z* 319.0894 (calc. C_19_H_14_N_2_OS [M + H]^+^, *m*/*z*: 319.0900).

*General procedure for reduction in nitriles***8a**,**b**. A mixture of corresponding nitrile **8** (1 eq.), 10% Pd/C (5–10 mol. %), and HCl_conc_ (1.5 eq.) in EtOH (3 mL) was placed into a glass autoclave, and hydrogen was passed through (p = 5 atm) while stirring until complete consumption of the starting nitrile (TLC control). The reaction mixture was filtered through celite, washed with EtOH, and the solvent was distilled off.

*General procedure for reduction in nitriles***8a**,**b**. A mixture of corresponding nitrile **8** (1 eq.), 10% Pd/C (5–10 mol. %), and HCl_conc_ (1.5 eq.) in EtOH (3 mL) was placed into a glass autoclave, and hydrogen was passed through (p = 5 atm) while stirring until complete consumption of the starting nitrile (TLC control). The reaction mixture was filtered through celite, washed with EtOH, and the solvent was distilled off.

*N-(5-(Aminomethyl)indan-2-yl)spiro[1,3-benzodioxole-2,1′-cyclohexane]-5-carboxamide (***9a***) hydrochloride.* This yielded pure N-(5-(aminomethyl)indan-2-yl)spiro[1,3-benzodioxole-2,1′-cyclohexane]-5-carboxamide **9a** hydrochloride (83%) as a light brown solid. mp: 209–211 °C. ^1^H NMR (CD_3_OD), δ: 1.53 (m, 2H), 1.74 (m, 4H), 1.91 (dist. t, 2H), 3.02 (m, 2H), 3.34 (m, 2H), 4.08 (s, 2H), 4.78 (m, 1H), 6.77 (d, 1H, ^3^J 8.3 Hz), 7.23–7.37 (m, 5H), 7.59 (s, 1H), 8.93 (br. s, 1H). ^13^C NMR (CD_3_OD), δ: 22.46, 23.72, 34.33, 38.02, 38.26, 42.87, 51.07, 106.69, 106.99, 119.14, 121.25, 124.50, 124.58, 126.96, 127.00, 131.04, 141.87, 141.96, 147.17, 149.93, 167.90. HRMS (ESI), *m*/*z* 379.2011 (calc. C_23_H_26_N_2_O_3_ [M + H]^+^, *m*/*z*: 379.2016).

*N-(5-(aminomethyl)indan-2-yl)-4,5,6,7-tetrahydrobenzo[c]*[1,2,5]*oxadiazole-5-carboxamide (**9b**) hydrochloride.* This yielded pure N-(5-(aminomethyl)indan-2-yl)-4,5,6,7-tetrahydrobenzo[c][1,2,5]oxadiazole-5-carboxamide **9b** hydrochloride (83%) as a brown solid. mp: 240–241 °C (decomp.). ^1^H NMR (CD_3_OD), δ: 1.95 (m, 1H), 2.12 (m, 1H), 2.79–3.03 (m, 7H), 3.28 (m, 2H), 4.08 (s, 2H), 4.60 (m, 1H), 7.25–7.33 (m, 3H). ^13^C NMR (CD_3_OD), δ: 18.03, 21.98, 25.49, 38.27, 38.50, 38.99, 42.83, 50.37, 124.58, 124.65, 127.00, 131.11, 141.74, 141.79, 150.77, 150.95, 174.21. HRMS (ESI), *m*/*z* 313.1661 (calc. C_17_H_20_N_4_O_2_ [M + H]^+^, *m*/*z*: 313.1659).

*N-(5-(Aminomethyl)indan-2-yl)benzo[b]thiophene-5-carboxamide (***9c***).* A solution of N-(5-cyanoindan-2-yl)benzo[b]thiophene-5-carboxamide **8c** (172 mg, 0.54 mmol) in absolute methanol (20 mL) was cooled to 0 °C. Then, CoCl_2_·6H_2_O (257 mg, 1.08 mmol) and NaBH_4_ (204 mg, 5.40 mmol) were added under an inert atmosphere (argon). The reaction mixture was stirred under cooling at 0 °C for 30 min and then at room temperature for 4 h. After that, distilled water (2 mL) and 20% aqueous solution of KOH were added to the reaction mixture (pH = 9). The precipitate formed was filtered off and washed with methanol. The solvent was distilled off from the combined filtrate. The product was dissolved in distilled water, and the solution was extracted with CH_2_Cl_2_. The combined organic phases were dried over anhydrous Na_2_SO_4_, then filtered, and the solvent was distilled off. The resulting product was purified by column chromatography (eluents CHCl_3_, CHCl_3_/EtOH (100:1), CHCl_3_/EtOH (50:1), CHCl_3_/EtOH (20:1), CHCl_3_/EtOH (5:1)). This yielded 80 mg (0.25 mmol, 46%) of pure N-(5-(aminomethyl)indan-2-yl)benzo[b]thiophene-5-carboxamide **9c** as a white solid. mp: 223–225 °C. ^1^H NMR (CD_3_OD), δ: 2.95–3.06 (m, 2H), 3.27–3.37 (m, 2H), 3.80 (s, 2H), 4.84 (m, 1H), 7.15 (d, 1H, ^3^J 7.5 Hz), 7.19–7.23 (m, 2H), 7.44 (d, 1H, ^3^J 5.5 Hz), 7.65 (d, 1H, ^3^J 5.5 Hz), 7.79 (dd, 1H, ^3^J 8.5 Hz, ^4^J 1.7 Hz), 7.96 (d, 1H, ^3^J 8.5 Hz), 8.32 (s, 1H). ^13^C NMR (CD_3_OD), δ: 37.99, 38.29, 44.47, 51.16, 121.58, 122.11, 122.18, 123.14, 123.52, 123.87, 125.54, 127.27, 130.18, 138.78, 139.06, 139.70, 141.10, 142.31, 168.68. HRMS (ESI), *m*/*z* 323.1208 (calc. C_19_H_18_N_2_OS [M + H]^+^, *m*/*z*: 323.1213).

*General procedure for alkylation of amines***9a,b,c**. To a solution of 3,7-bis(chloroacetyl)-1,5-dimethyl-3,7-diazabicyclo[3.3.1]nonan-9-one **10** (1 eq.) and the corresponding amine **9** (1 eq.) in NMP, K_2_CO_3_ (5 eq.) was added, and the resulting mixture was stirred under heating at 75 °C for 15 h. After that, the reaction mixture was cooled to room temperature, filtered off, and the solid residue was washed with NMP until no more product was washed out (TLC control). The filtrates were combined, and the solvent was distilled off with an oil pump. The solid residue was treated with Et_2_O and filtered off. The resulting product was purified by column chromatography (eluents CHCl_3_, CHCl_3_/EtOH (100:1), CHCl_3_/EtOH (50:1)).

*N-(5-((1,11-Dimethyl-4,8,12-trioxo-3,6,9-triazatricyclo[7.3.1.1^3,11^]tetradec-6-yl)methyl)indan-2-yl)spiro[1,3-benzodioxole-2,1′-cyclohexane]-5-carboxamide (***11a***).* This yielded pure N-(5-((1,11-dimethyl-4,8,12-trioxo-3,6,9-triazatricyclo[7.3.1.1^3,11^]tetradec-6-yl)methyl)indan-2-yl)spiro[1,3-benzodioxole-2,1′-cyclohexane]-5-carboxamide **11a** (63%) as a white solid. R_f_ = 0.43 (CHCl_3_/EtOH (20:1)). mp: 147–149 °C. ^1^H NMR (CDCl_3_), δ: 0.99 (s, 3H), 1.08 (s, 3H), 1.50 (m, 2H), 1.72 (m, 4H), 1.89 (m, 4H), 2.72 (d, ^2^J 13.5 Hz), 2.88–2.94 (m, 2H), 3.01 (d, 2H, ^2^J 13.2 Hz), 3.18–3.23 (m, 2H), 3.35–3.44 (m, 2H), 3.58 (s, 2H), 3.75–3.79 (m, 2H), 4.79 (m, 1H), 4.90–4.93 (m, 4H), 6.24 (d, 1H, ^3^J 7.3 Hz), 6.71 (d, 1H, ^3^J 8.1 Hz), 7.13–7.25 (m, 5H). ^13^C NMR (CDCl_3_), δ: 15.82, 16.21, 23.08, 24.40, 35.16, 39.79, 39.97, 45.36, 45.78, 51.22, 54.34, 55.43, 60.32, 60.45, 62.42, 107.25, 107.79, 120.01, 120.97, 125.10, 126.44, 127.76, 128.63, 134.45, 141.45, 141.86, 147.80, 150.36, 166.94, 168.15, 210.91. HRMS (ESI), *m*/*z* 627.3179 (calc. C_36_H_42_N_4_O_6_ [M + H]^+^, *m*/*z*: 627.3177).

*N-(5-((1,11-Dimethyl-4,8,12-trioxo-3,6,9-triazatricyclo[7.3.1.1^3,11^]tetradec-6-yl)methyl)indan-2-yl)-4,5,6,7-tetrahydrobenzo[c]*[1,2,5]*oxadiazole-5-carboxamide (***11b***).* This yielded pure N-(5-((1,11-dimethyl-4,8,12-trioxo-3,6,9-triazatricyclo[7.3.1.1^3,11^]tetradec-6-yl)methyl)indan-2-yl)-4,5,6,7-tetrahydrobenzo[c][1,2,5] oxadiazole-5-carboxamide **11b** (53%) as a white solid. R_f_ = 0.35 (CHCl_3_/EtOH (20:1)). mp: 169–171°C. ^1^H NMR (CDCl_3_), δ: 1.00 (s, 3H), 1.09 (s, 3H), 2.00 (m, 1H), 2.19 (m, 1H), 2.56 (m, 1H), 2.73–2.87 (m, 5H), 3.04 (d, 2H, ^2^J 13.6 Hz), 3.11–3.23 (m, 5H), 3.33–3.41 (m, 2H), 3.61 (s, 2H), 3.73–3.78 (m, 2H), 4.80 (m, 1H), 4.91 (m, 4H), 6.05 (d, 1H, ^3^J 7.2 Hz), 7.14–7.29 (m, 3H). ^13^C NMR (CDCl_3_), δ: 15.87, 16.22, 19.08, 23.29, 26.05, 39.83, 39.91, 40.50, 45.35, 45.82, 50.82, 54.34, 55.49, 60.18, 60.35, 62.34, 125.13, 126.45, 128.76, 134.36, 141.17, 141.58, 150.76, 150.99, 168.12, 172.70, 210.82. HRMS (ESI), *m*/*z* 561.2824 (calc. C_30_H_36_N_6_O_5_ [M + H]^+^, *m*/*z*: 561.2820).

*N-(5-((1,11-Dimethyl-4,8,12-trioxo-3,6,9-triazatricyclo[7.3.1.1^3,11^]tetradec-6-yl)methyl)indan-2-yl)benzo[b]thiophene-5-carboxamide (***11c***).* This yielded pure N-(5-((1,11-dimethyl-4,8,12-trioxo-3,6,9-triazatricyclo[7.3.1.1^3,11^]tetradec-6-yl)methyl)indan-2-yl)benzo[b]thiophene-5-carboxamide **11c** (56%) as a white solid. R_f_ = 0.31 (CHCl_3_/EtOH (20:1)). mp: 162–164 °C. ^1^H NMR (CDCl_3_), δ: 1.00 (s, 3H), 1.09 (s, 3H), 2.74 (d, 2H, ^2^J 13.5 Hz), 2.96–3.03 (m, 4H), 3.21–3.26 (m, 2H), 3.44–3.51 (m, 2H), 3.60 (s, 2H), 3.78 (d, 2H, ^2^J 14.2 Hz), 4.93 (d, 4H, ^2^J 13.5 Hz), 5.02 (m, 1H), 6.45 (d, 1H, ^3^J 6.6 Hz), 7.17 (d, 1H, ^3^J 7.7 Hz), 7.22 (s, 1H), 7.30 (d, 1H, ^3^J 7.7 Hz), 7.40 (d, 1H, ^3^J 5.6 Hz), 7.53 (d, 1H, ^3^J 5.6 Hz), 7.71 (dd, 1H, ^3^J 8.4 Hz, ^4^J 1.5 Hz), 7.92 (d, 1H, ^3^J 8.4 Hz), 8.25 (d, 1H, ^4^J 1.5 Hz). ^13^C NMR (CDCl_3_), δ: 15.84, 16.21, 39.87, 40.03, 45.37, 45.83, 51.35, 54.34, 55.45, 60.34, 60.43, 62.44, 122.42, 122.57, 122.61, 124.19, 125.15, 126.49, 127.98, 128.69, 130.73, 134.53, 139.44, 141.42, 141.84, 142.72, 167.58, 168.15, 210.94. HRMS (ESI), *m*/*z* 571.2369 (calc. C_32_H_34_N_4_O_4_S [M + H]^+^, *m*/*z*: 571.2374).

### 3.2. Radioligand Binding

*Synthesis of Tritium-Labeled PAM-43.* Tritium-labeled PAM-43 (3,7-bis(2,3-dihydro-1-benzofuran-5-ylcarbonyl)-1,5-dimethyl-3,7-diazabicyclo[3.3.1]-nonan-9-one) was synthesized at Laboratory of Molecular Pharmacology of Peptides, Institute of Molecular Genetics, National Research Centre «Kurchatov Institute» (Moscow, Russia). A solid-phase method [56] was used. The resulting compound [^3^H]PAM-43 was characterized by the specific activity of 132 Ci/mmol and a chemical purity of 98.7%.

*Radioligand Binding.* The radioligand-receptor assay was performed on a special unit, and the MultiScreenHTS 96-Well Filter Plates (MultiScreen System, EMD Millipore, Darmstadt, Germany) were used. As a main radioligand, we used tritium-labeled PAM-43 ([^3^H]PAM-43 with a specific radioactivity of 132 Ci/mmol and radiochemical purity of over 95%). Plasmatic membranes of rat brain cells were the target tissue. The incubation of the reaction mixture was performed directly in the wells of the standard 96-well plates with GF/B filters (Millipore). The reaction mixture (final volume, 200 μL) contained: 50 μL of the radioactively labeled ligand in buffer solution and 50 μL of buffer (containing either unlabeled ligand PAM-43 (250 μM), or the compound under study, depending on the experiment point), and a membrane protein solution. The reaction was initiated by the addition of 100 μL of membrane protein solution (whose final concentration in the incubation mixture was 0.2 mg/mL) dissolved in Buffer B (50 mM Tris-HCl, 1 mM CaCl_2_, 0.003% BSA, pH 7.4 at 30 °C for incubation or pH 7.4 at 4 °C for plates washing) with an added cocktail of inhibitors (100 μM PMSF + 10 μM Bacitracin + 5 μM Pepstatin A). In some experiments, the reaction mixture contained 250 μM of Glu. L-Glu was preliminarily added to plasmatic membranes in the concentration of 500 μM (30 min before the incubation with the radioligand). The plates were kept at 30 °C with continuous shaking for 20 min. After the incubation, the plates were air-dried, filters were detached, and transferred into scintillation vials, each containing 4 mL of the liquid scintillator (Unisolve 100; Koch-Light, Haverhill, UK); radioactivity was measured using a Tri-Carb 2100R liquid scintillation counter (Packard BioScience, USA). Mathematical processing of results was conducted using the SigmaPlot 10.0 software suite (Systat Software Inc., San Jose, CA, USA).

*Experimental design.* There were three types of the reaction mixture on a standard 96-well plate. The first type contained the radioactively labeled ligand and a membrane protein solution. The second type contained the radioactively labeled ligand, a membrane protein solution, and the unlabeled ligand PAM-43. Additionally, the third type contained the radioactively labeled ligand, a membrane protein solution, and the compound under study (in different concentrations). Specific binding was determined as the difference in radioactivity measured in samples of the first and second types. A value of 100% on the graphs corresponds to [^3^H]PAM-43 specific binding determined in the presence of unlabeled PAM-43. The data measured in the samples of the third type were subjected to statistical and mathematical processing, taking into account the value of nonspecific binding. The influence of the compounds under study on [^3^H]PAM-43-specific binding was represented as a proportion (in %) of [^3^H]PAM-43-specific binding.

*Statistical Analysis.* Data from the radioligand-receptor binding assay were determined using non-linear regression analysis (five-parameter logistic curve) included in the Pharmacology module of the SigmaPlot 10 software suite (Systat Software Inc., San Jose, CA, USA). Values on the graphs represent the mean ± S.E. of 3 independent experiments. Each experiment was conducted on isolated plasmatic membranes of different groups of rats; each mean in the experiment was obtained as average from 6 separated experimental volumes (wells of the standard 96-well plates with GF/B filters (Millipore)).

*Animal Management.* Male adult albino outbred rats (mean body weight, 180–200 g) were housed in plastic cages under standard laboratory conditions, which included a controlled ambient temperature (22–25 °C), a 12 h light/dark cycle, and 60 ± 10% humidity during all experiments. Before the start of an experiment, animals were allowed access to standard laboratory rat pellet chow and water ad libitum.

*Isolation of Plasma Membranes.* Rat brain cells’ plasmatic membranes were isolated at 4 °C. The rats were decapitated; their brains were washed with cold PBS, and brain structures (cortex and hippocampus) were isolated and added to Buffer A1 (10 mM Tris-HCl, pH 7.4 at 4 °C, saccharose 0.32 M, 1 mM EDTA, 1 mM benzamidine, 0.1 mM PMSF). The resulting samples were homogenized in 10 volumes of the buffer using a Teflon-in-glass homogenizer, and then the homogenate was centrifuged at 1000× *g* for 20 min, the sediment removed, and the supernatant centrifuged at 40,000× *g* for 30 min. The dense brown mitochondria-rich sediment at the bottom of the tube was removed, and the less-dense translucent sediment of membranes was resuspended in Buffer A1, transferred to a clean tube, and centrifuged again at 40,000× *g* for 30 min. The sediment was resuspended in Buffer A2 (10 mM Tris-HCl, pH 7.4 at 4 °C, 0.22 M saccharose), divided into portions, frozen in liquid nitrogen, and stored no longer than 30 days at −70 °C. Protein concentration in membrane samples was measured according to the Hartree–Lowry method.

## 4. Conclusions

In this paper, we described the development and optimization of synthetic approaches to three novel compounds designed as potential allosteric modulators of AMPA receptors. For the synthesized compounds, the radioligand-receptor binding analysis was performed using [^3^H]PAM-43, a synthetic positive allosteric modulator of AMPA receptors described earlier, as a standard ligand. According to the published data, there exist multiple binding sites in the AMPA receptor. The simultaneous action of a ligand on several sites can change the conformation of the receptor and lead to the net effect of either positive or negative allosteric modulation.

As a result of radioligand-receptor binding analysis, it was found that two synthesized compounds **11b** and **11c** bind with the same targets as positive allosteric modulator PAM-43 (at least on AMPA receptors) but with different binding sites, leading to a significant increase in the [^3^H]PAM-43 response. For compound **11a** our results may indicate the selectivity with respect to the PAM-43 binding site, but its effect is weaker. We also hypothesize that the enhancement of radioligand binding may be due to specific binding cooperativity effects, and further optimization of the structures described here can lead to broad-spectrum drug candidates for the treatment and prevention of diseases associated with impaired functions of the central nervous system.

## Data Availability

The data of the current study are available from the corresponding author upon reasonable request.

## Supplementary Materials

The following supporting information can be downloaded at: https://www.mdpi.com/article/10.3390/ijms241210293/s1, ^1^H NMR and ^13^C NMR spectra for all isolated compounds.
