# Supplementary material for: New Allosteric Modulators of AMPA Receptors: Synthesis and Study of Their Functional Activity by Radioligand-Receptor Binding Analysis
Source: Int J Mol Sci. 2023 Jun 18;24(12):10293. doi: 10.3390/ijms241210293 (PMC10299041; doi:10.3390/ijms241210293)

SUPPLEMENTARY MATERIALS

to the article

New Allosteric Modulators of AMPA Receptors: Synthesis and Study of Their Functional Activity by Radioligand-Receptor Binding Analysis

Elena A. Golubeva [1], Mstislav I. Lavrov [1], Polina N. Veremeeva [1], Tatiana V. Vyunova [2], Konstantin V. Shevchenko [2], Maxim A. Topchiy [3], Andrey F. Asachenko [3] and Vladimir A. Palyulin [1],*

[1] Department of Chemistry, Lomonosov Moscow State University, 119991 Moscow, Russia
[2] Laboratory of Molecular Pharmacology of Peptides, Institute of Molecular Genetics, National Research Centre Kurchatov Institute, 123182 Moscow, Russia
[3] A.V. Topchiev Institute of Petrochemical Synthesis, Russian Academy of Sciences, 119991 Moscow, Russia
* Correspondence: vap@qsar.chem.msu.ru

NMR spectra

^{1}H NMR N-(5-cyanoindan-2-yl)spiro[1,3-benzodioxole-2,1'-cyclohexane]-5-carboxamide (**8a**)

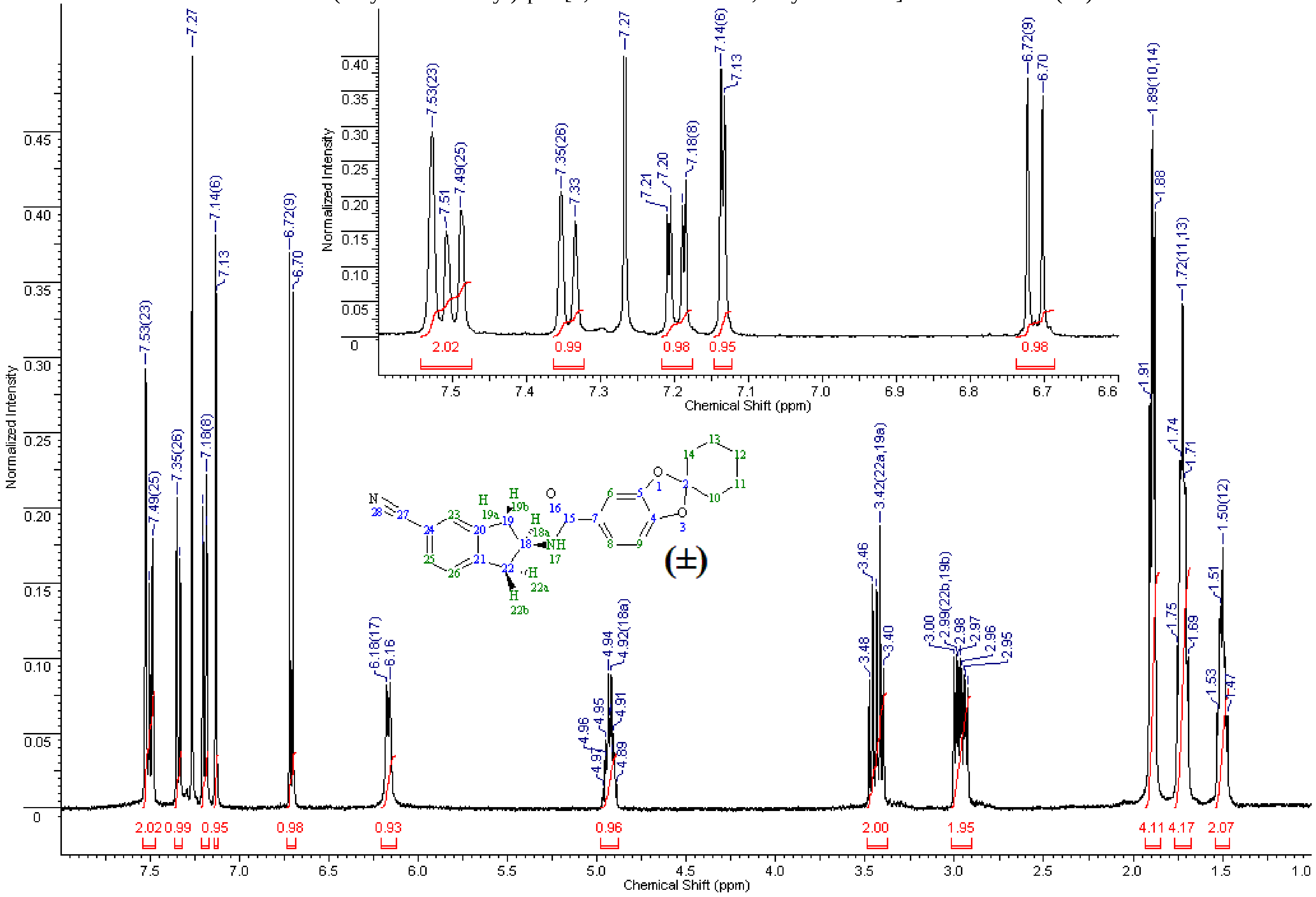

^{13}C NMR N-(5-cyanoindan-2-yl)spiro[1,3-benzodioxole-2,1'-cyclohexane]-5-carboxamide (**8a**)

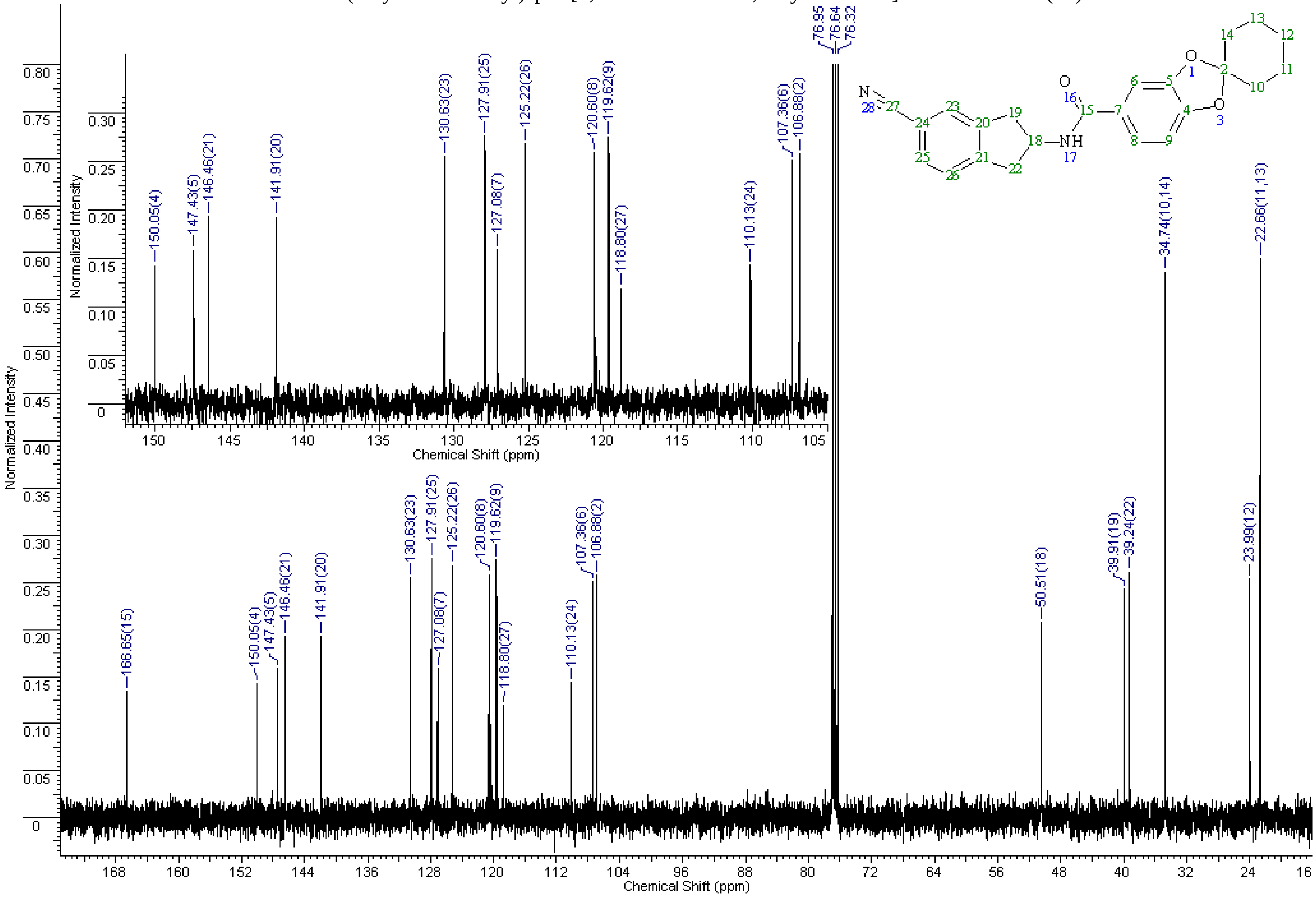

^{1}H NMR N-(5-cyanoindan-2-yl)benzo[c][1,2,5]oxadiazole-5-carboxamide (**8b**)

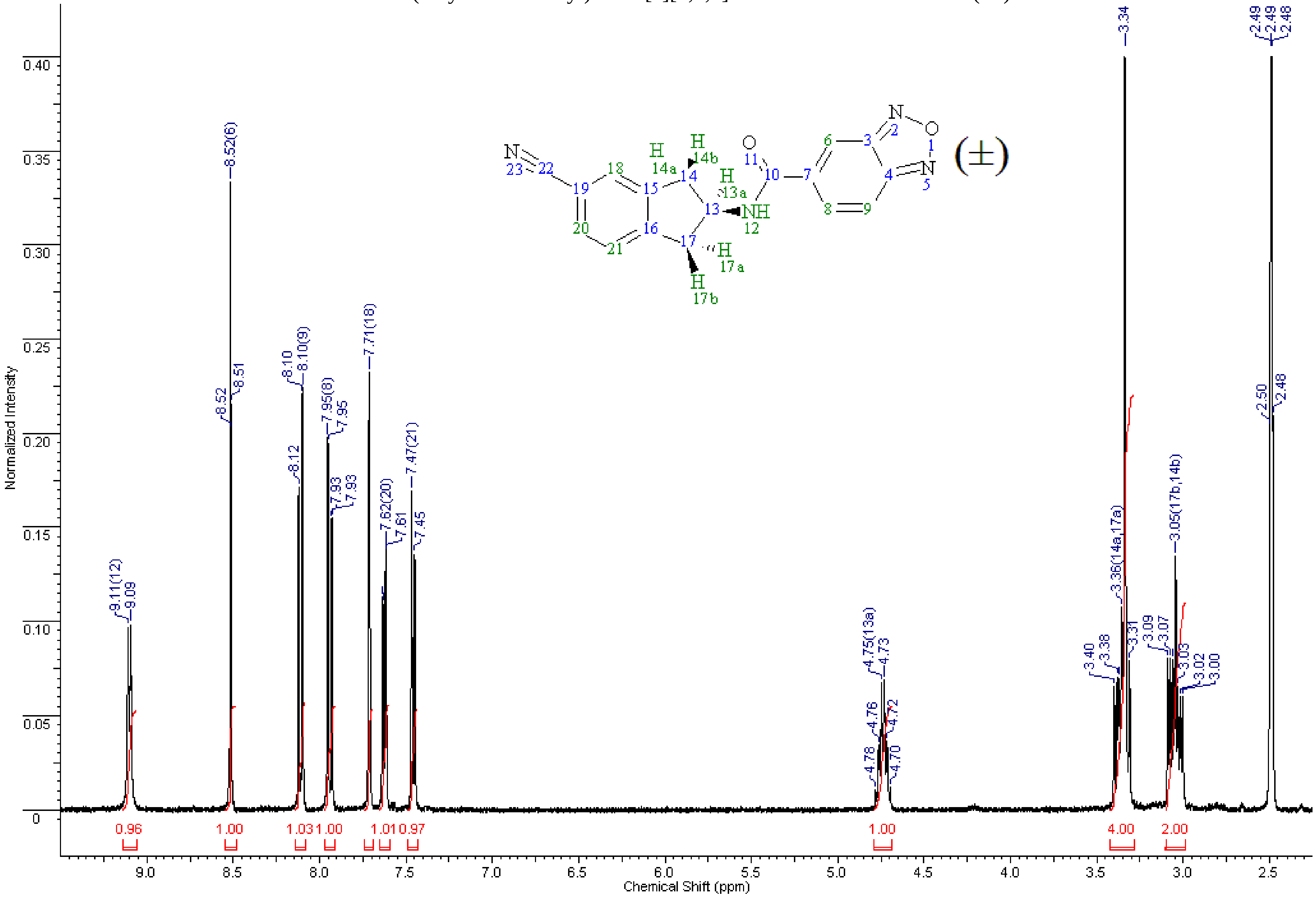

^{13}C NMR N-(5-cyanoindan-2-yl)benzo[c][1,2,5]oxadiazole-5-carboxamide (**8b**)

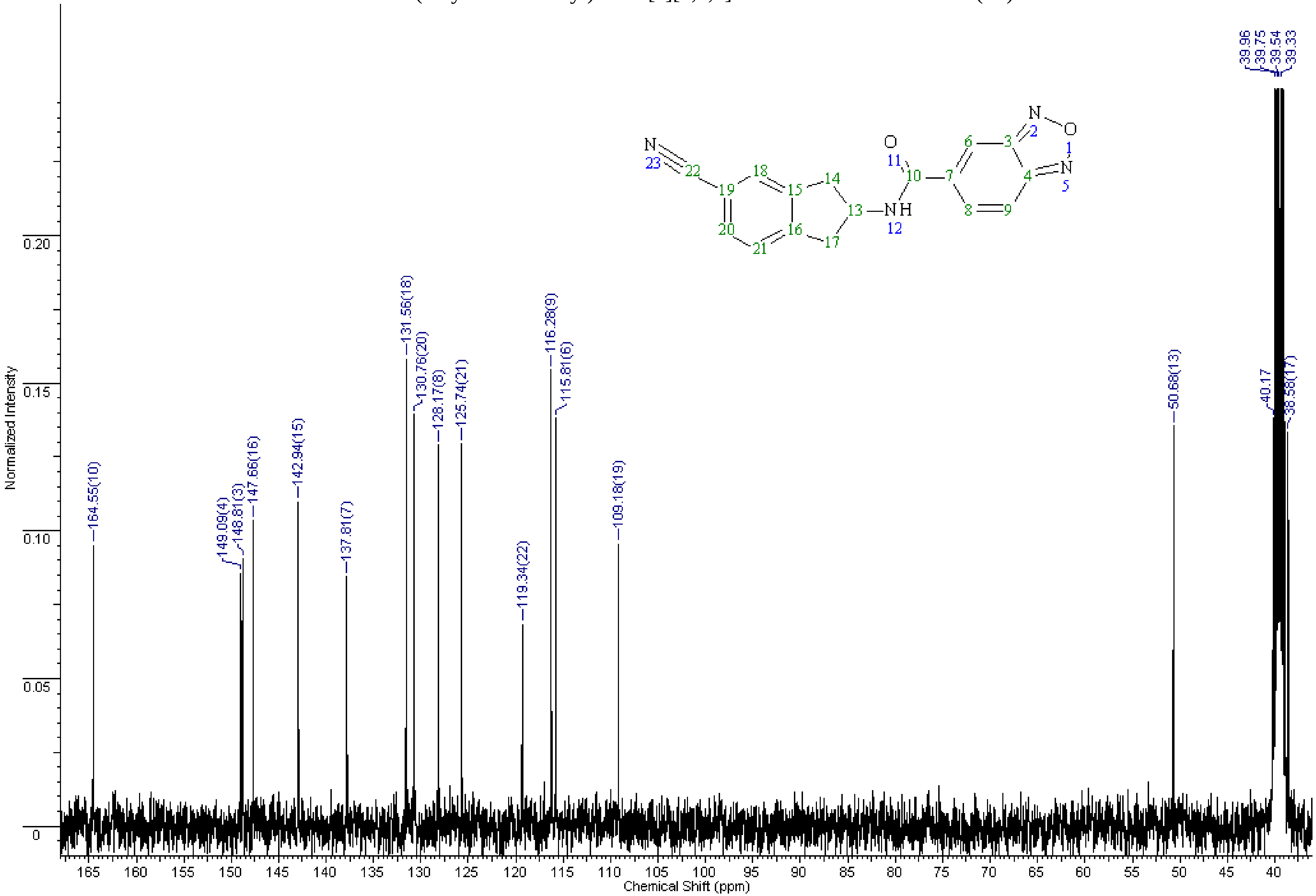

^{1}H NMR N-(5-cyanoindan-2-yl)benzo[b]thiophene-5-carboxamide (**8c**)

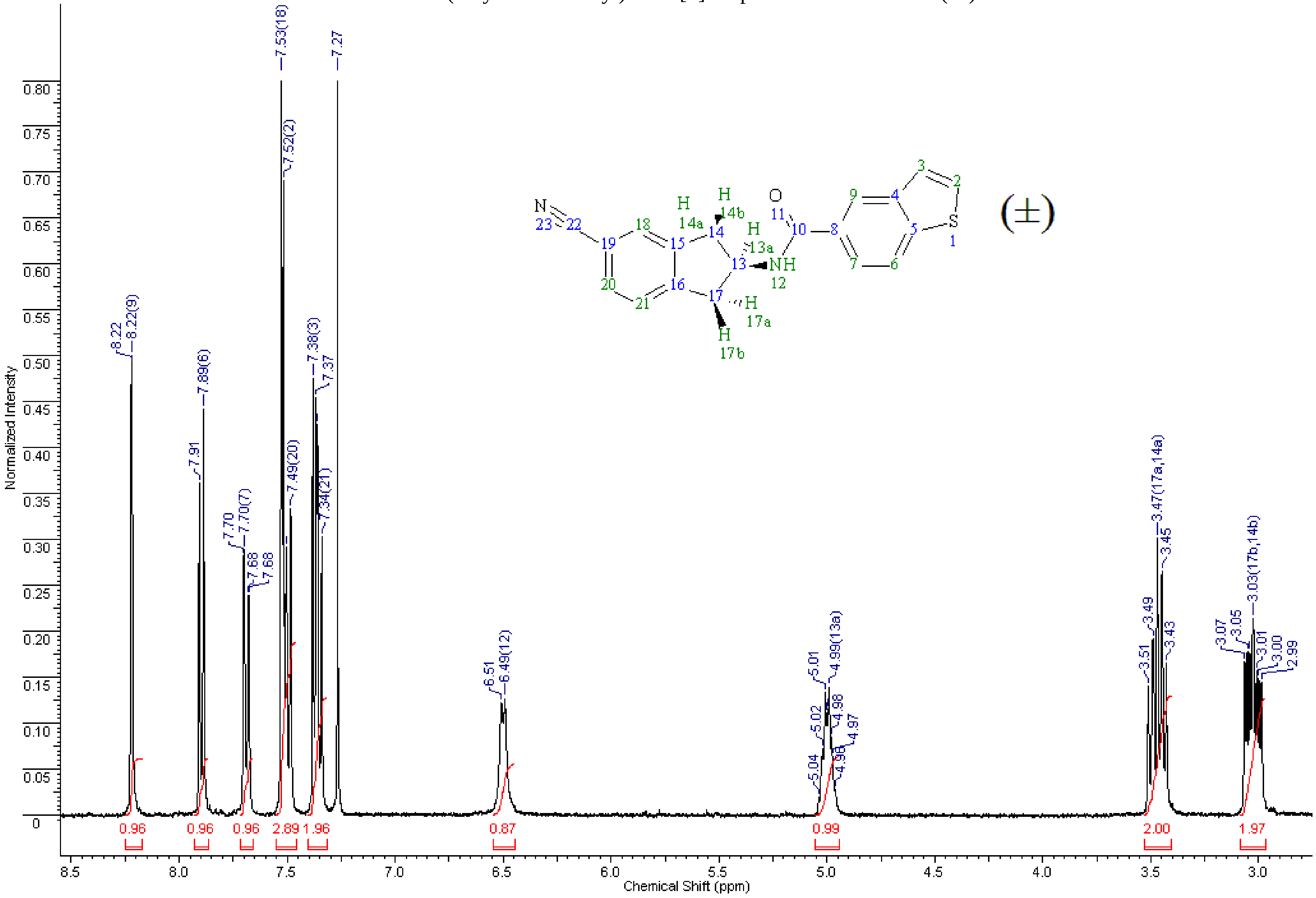

^{13}C NMR N-(5-cyanoindan-2-yl)benzo[b]thiophene-5-carboxamide (**8c**)

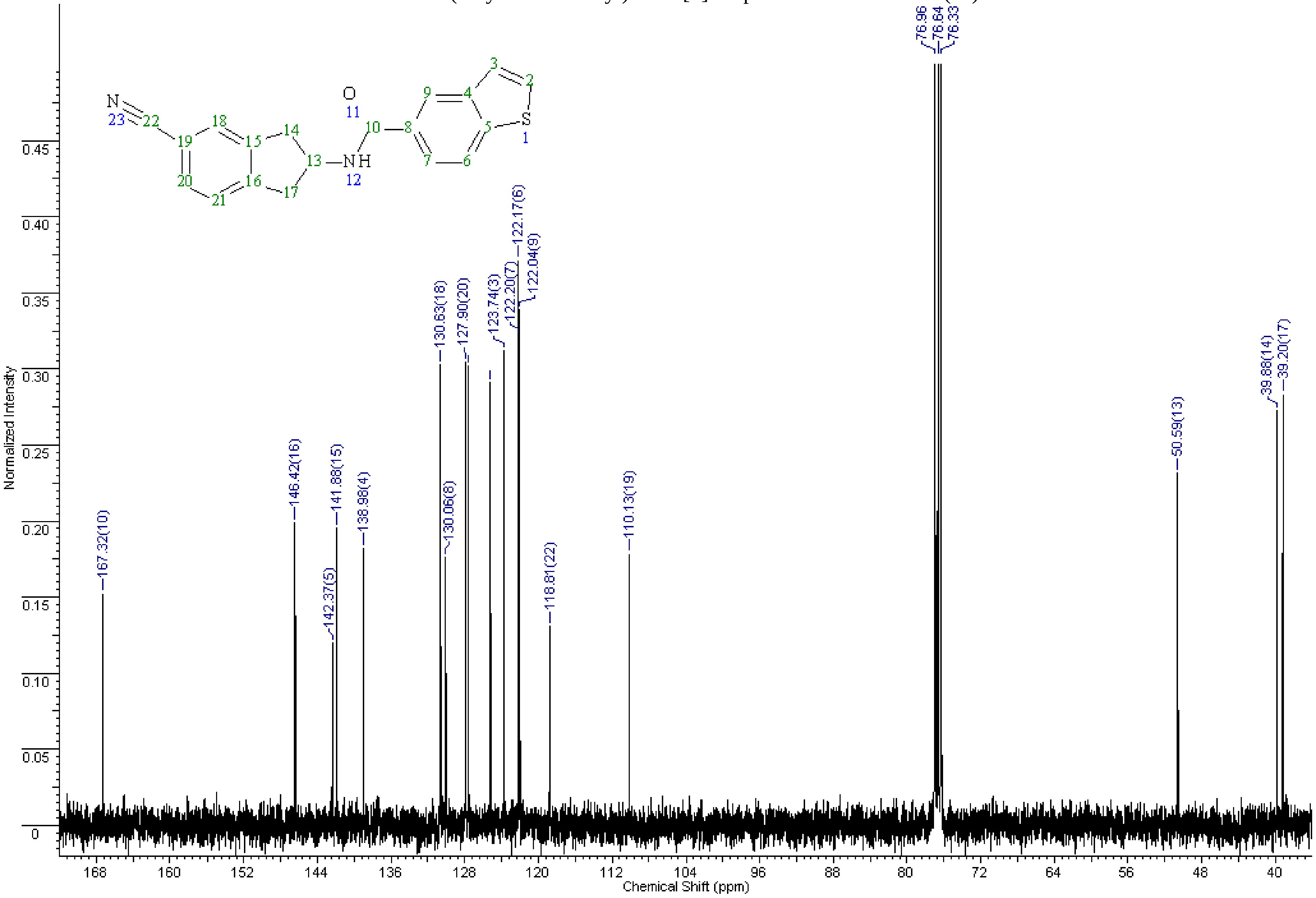

^{1}H NMR N-(5-(aminomethyl)indan-2-yl)spiro[1,3-benzodioxole-2,1'-cyclohexane]-5-carboxamide (**9a**) hydrochloride

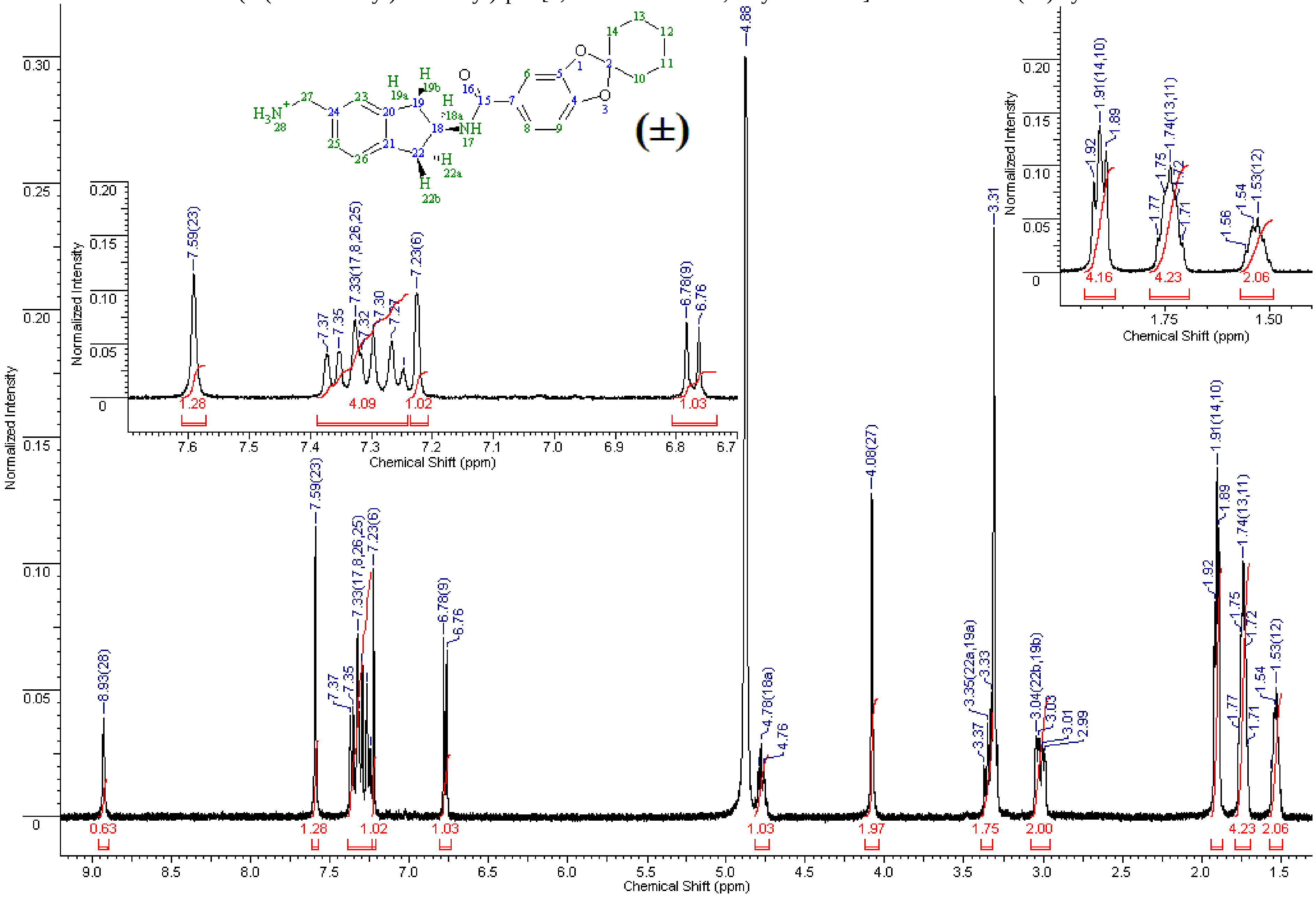

^{13}C NMR N-(5-(aminomethyl)indan-2-yl)spiro[1,3-benzodioxole-2,1'-cyclohexane]-5-carboxamide (**9a**) hydrochloride

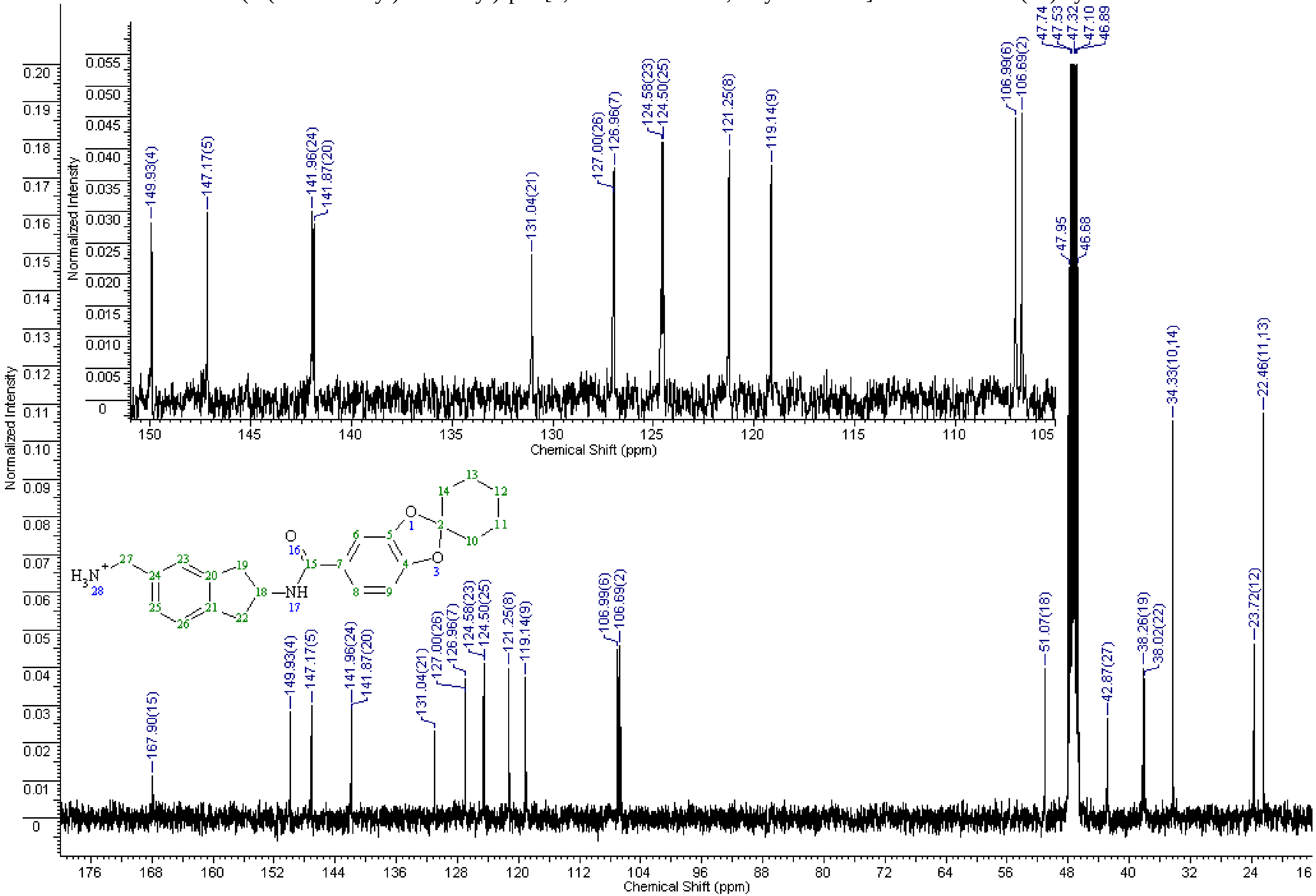

^{1}H NMR N-(5-(aminomethyl)indan-2-yl)-4,5,6,7-tetrahydrobenzo[c][1,2,5]oxadiazole-5-carboxamide (**9b**) hydrochloride

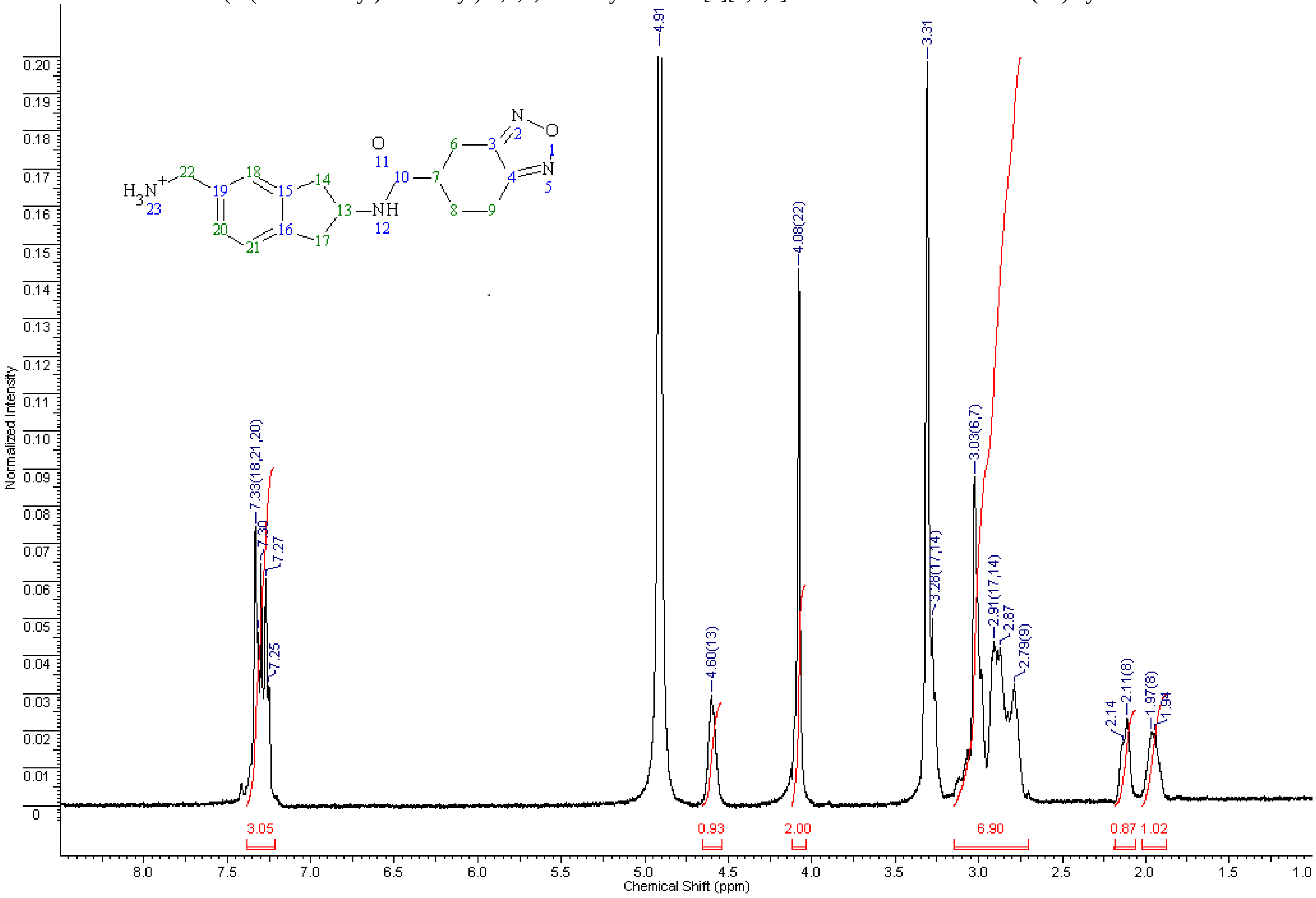

^{13}C NMR N-(5-(aminomethyl)indan-2-yl)-4,5,6,7-tetrahydrobenzo[c][1,2,5]oxadiazole-5-carboxamide (**9b**) hydrochloride

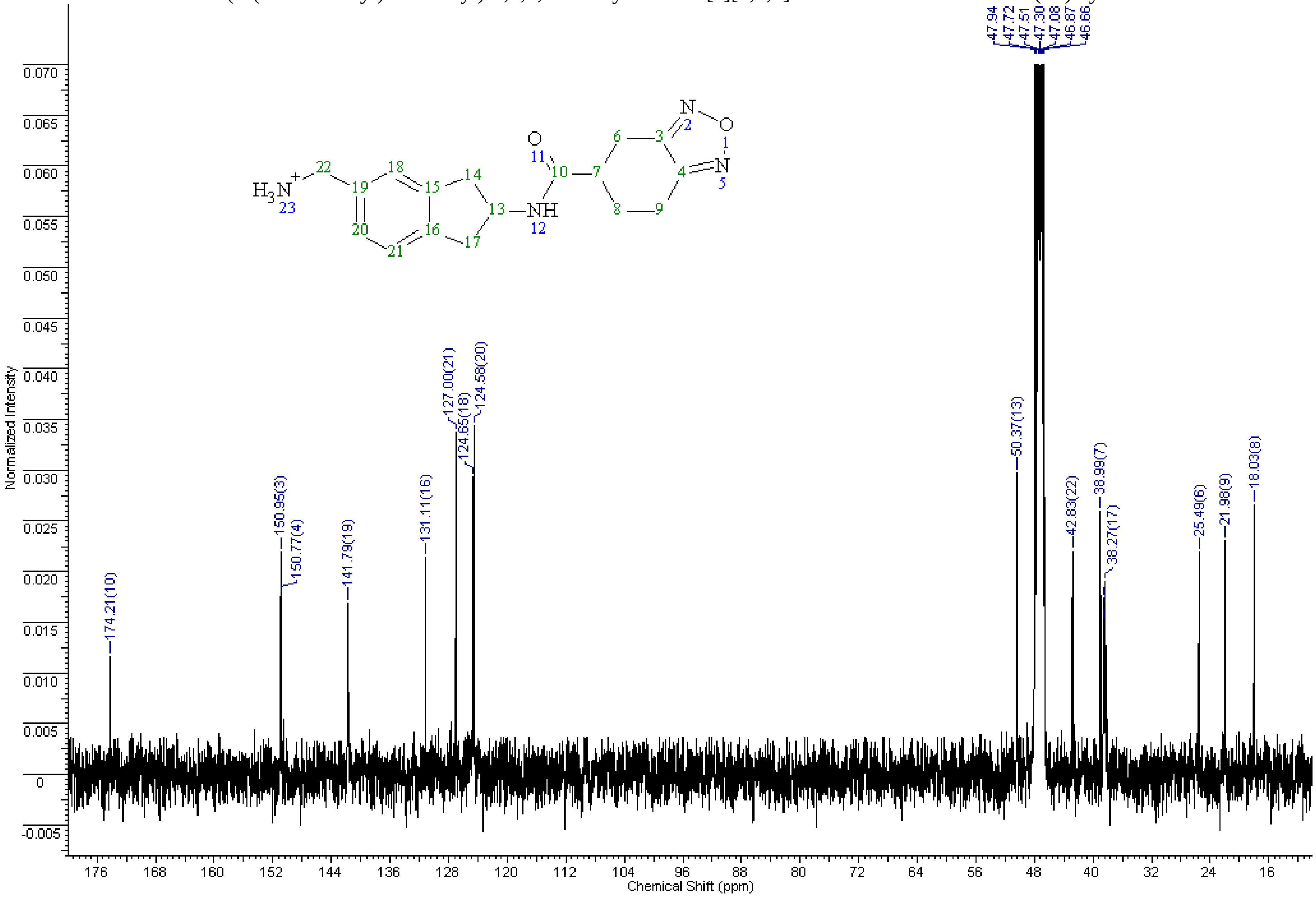

^{1}H NMR N-(5-(aminomethyl)indan-2-yl)benzo[b]thiophene-5-carboxamide (**9c**)

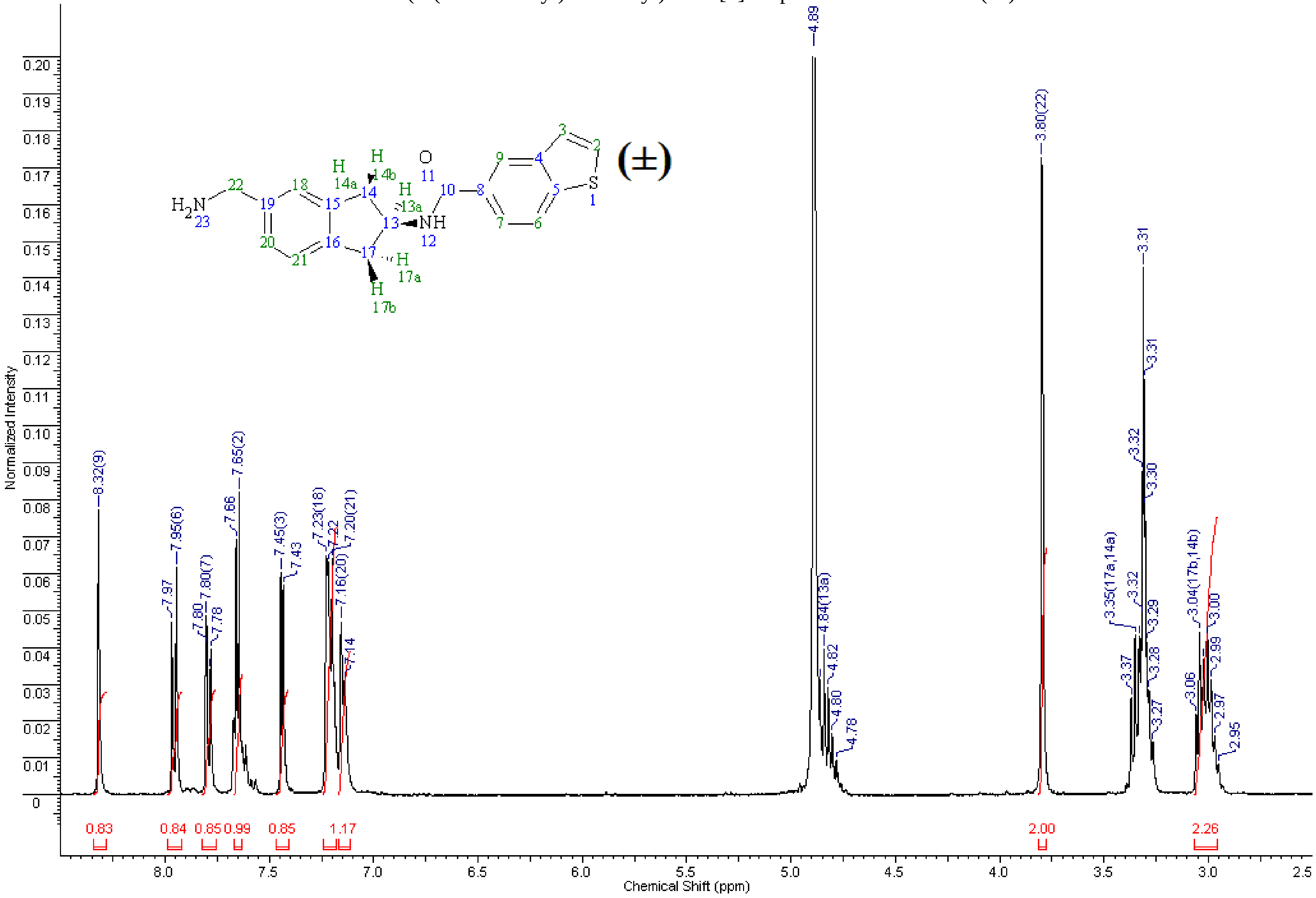

^{13}C NMR N-(5-(aminomethyl)indan-2-yl)benzo[b]thiophene-5-carboxamide (**9c**)

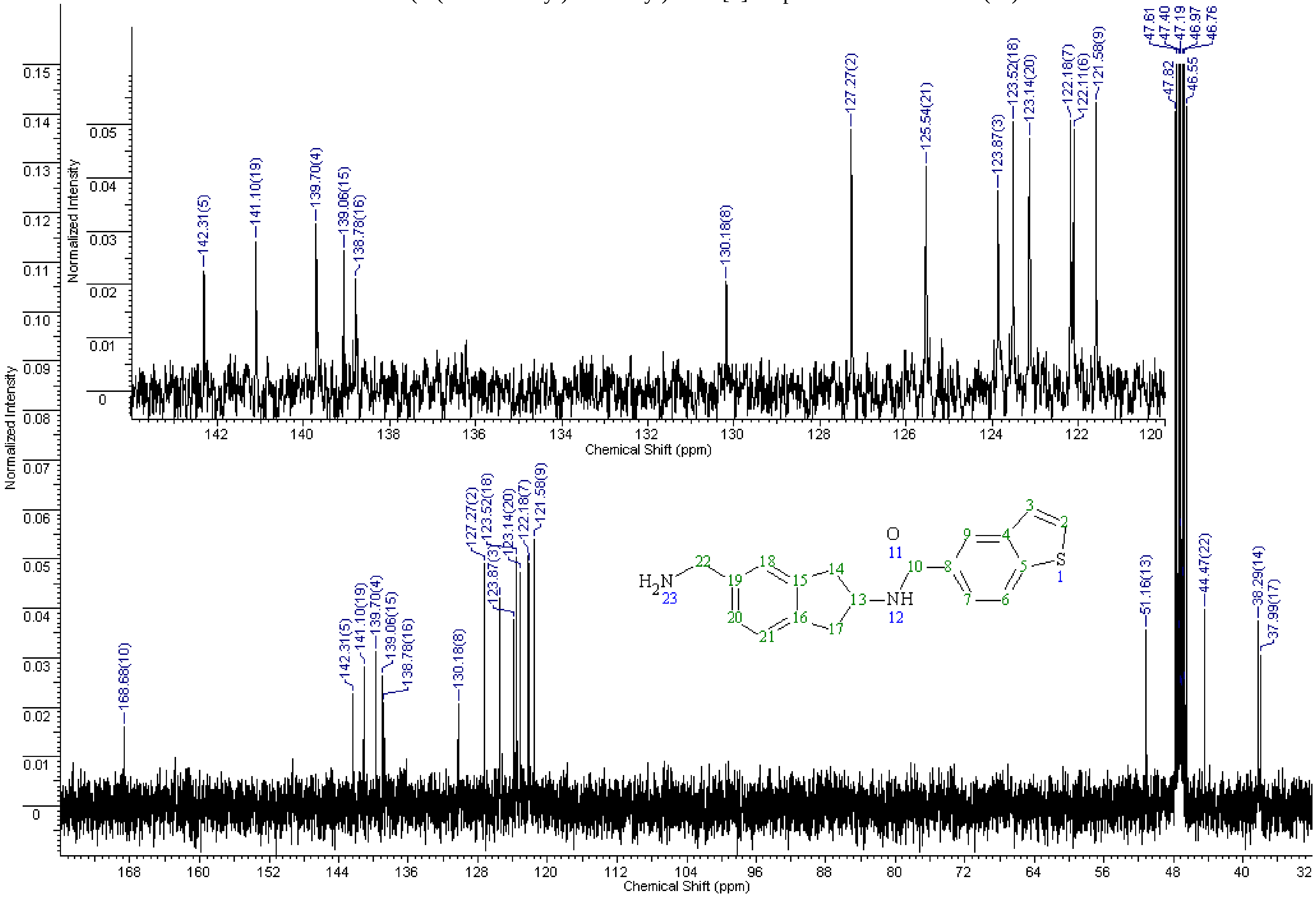

^{1}H NMR N-(5-((1,11-dimethyl-4,8,12-trioxo-3,6,9-triazatricyclo[7.3.1.1^{3,11}]tetradec-6-yl)methyl)indan-2-yl)spiro[1,3-benzodioxole-2,1'-cyclohexane]-5-carboxamide (**11a**)

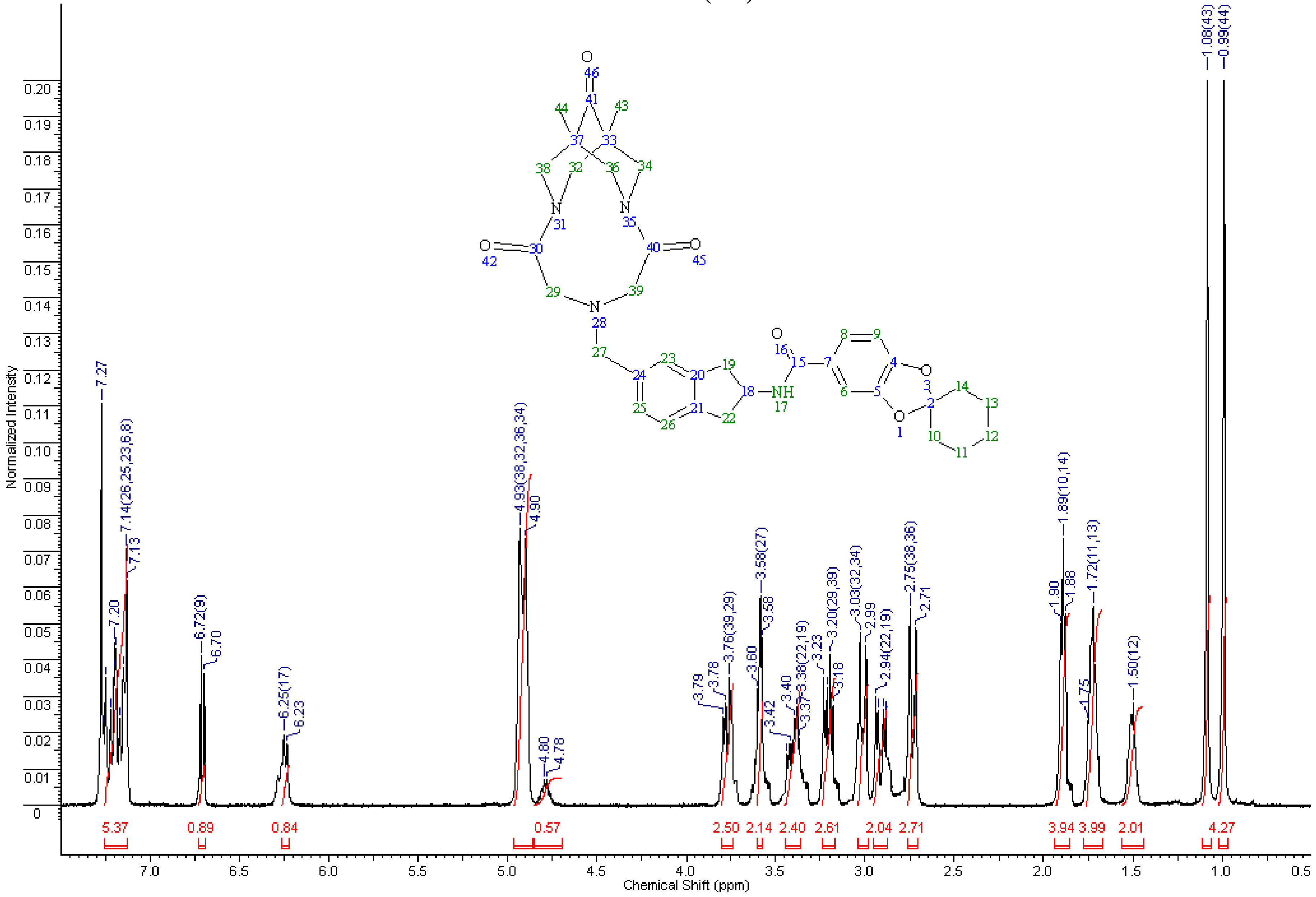

^{13}C NMR N-(5-((1,11-dimethyl-4,8,12-trioxo-3,6,9-triazatricyclo[7.3.1.1^{3,11}]tetradec-6-yl)methyl)indan-2-yl)spiro[1,3-benzodioxole-2,1'-cyclohexane]-5-carboxamide (**11a**)

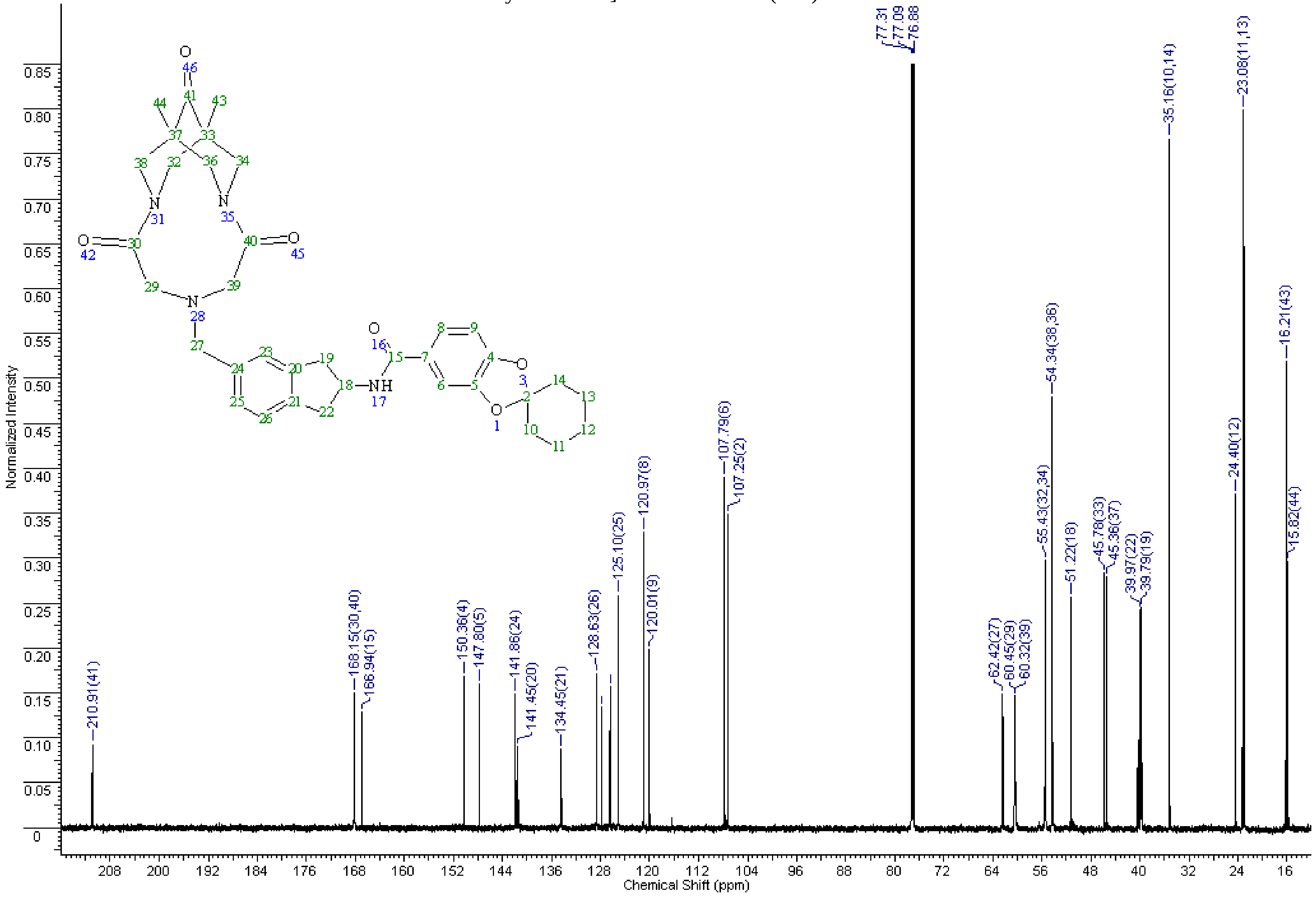

^{1}H NMR N-(5-((1,11-dimethyl-4,8,12-trioxo-3,6,9-triazatricyclo[7.3.1.1^{3,11}]tetradec-6-yl)methyl)indan-2-yl)-4,5,6,7-tetrahydrobenzo[c][1,2,5]oxadiazole-5-carboxamide (**11b**)

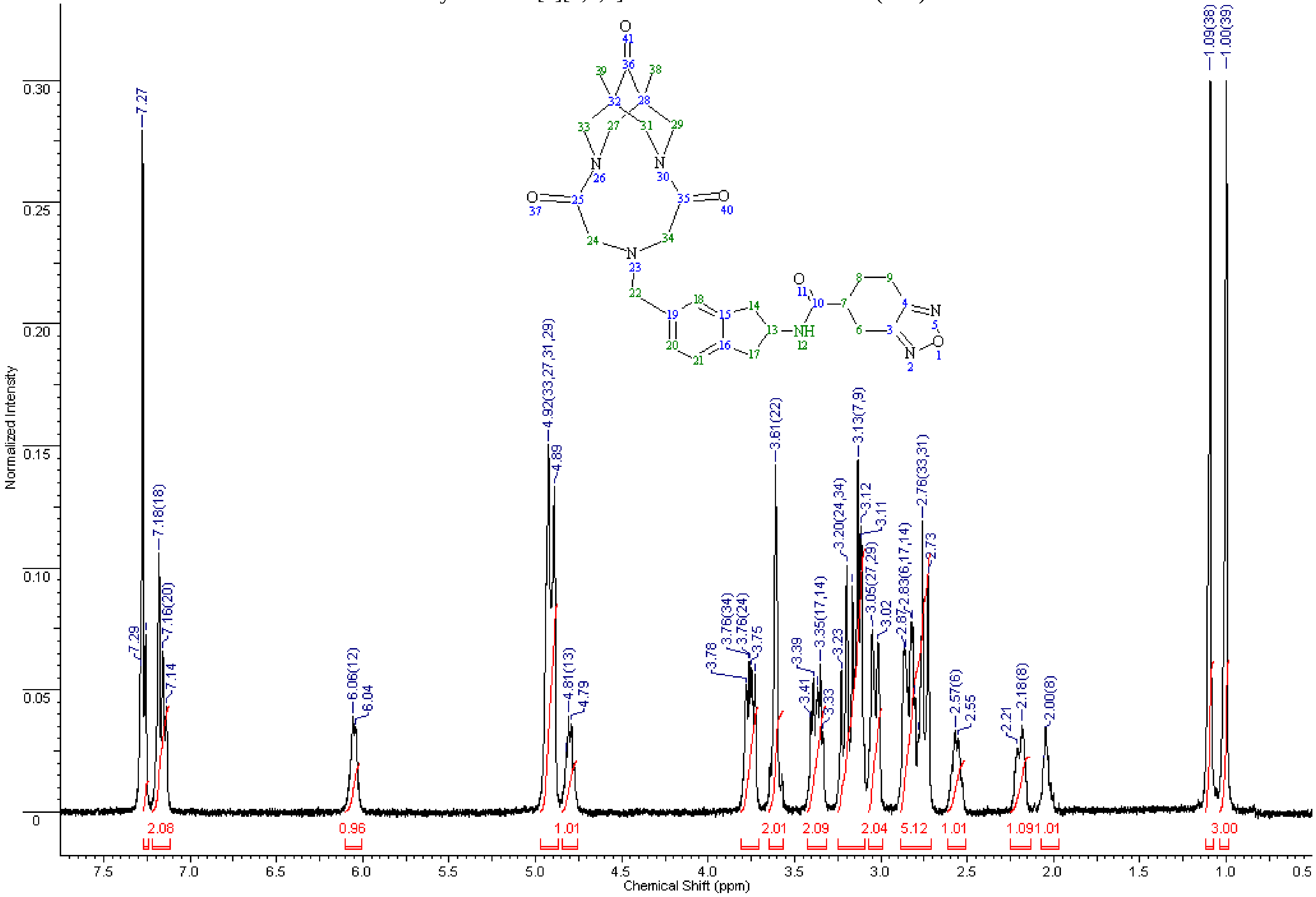

^{13}C NMR N-(5-((1,11-dimethyl-4,8,12-trioxo-3,6,9-triazatricyclo[7.3.1.1^{3,11}]tetradec-6-yl)methyl)indan-2-yl)-4,5,6,7-tetrahydrobenzo[c][1,2,5]oxadiazole-5-carboxamide (**11b**)

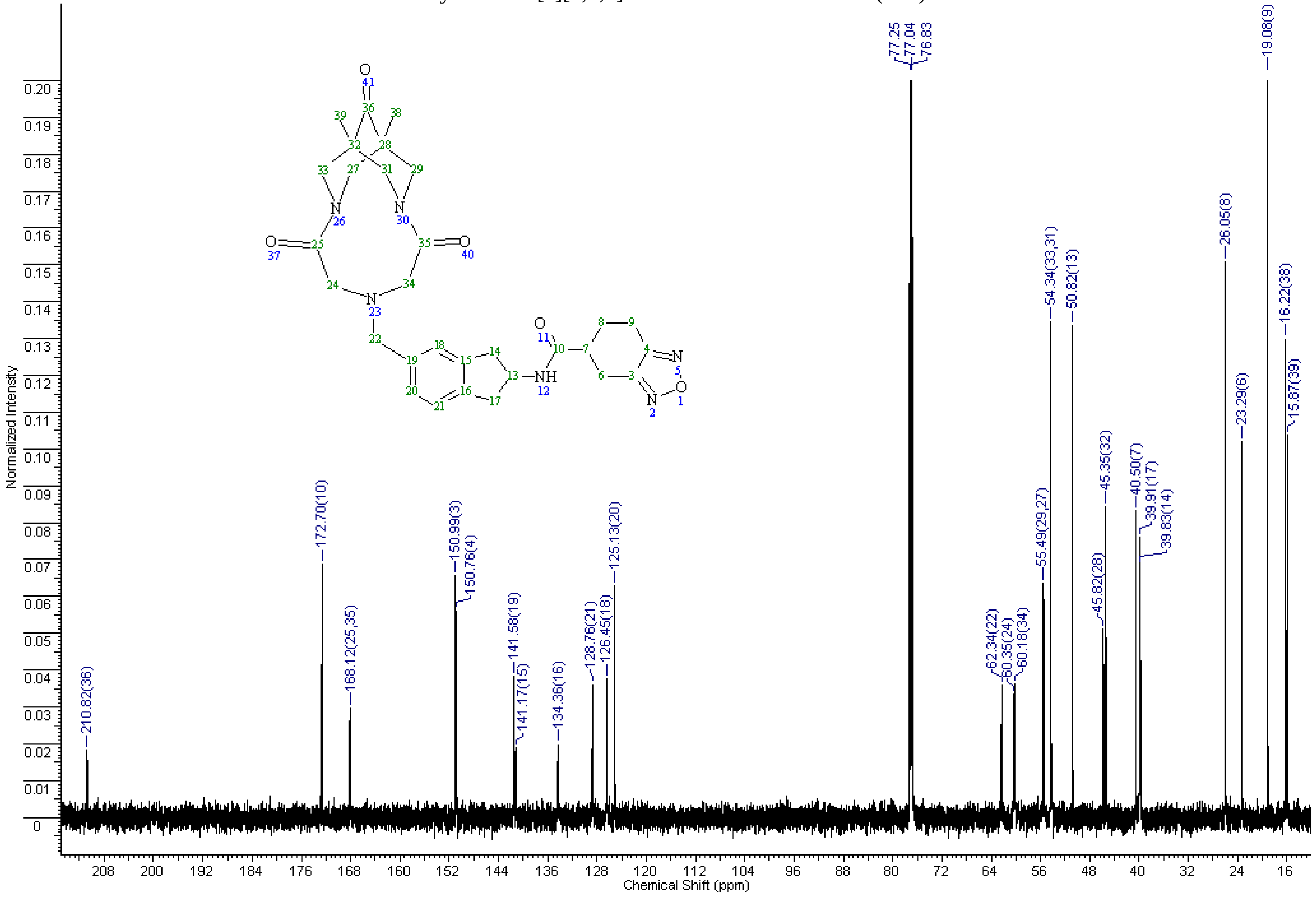

^{1}H NMR N-(5-((1,11-dimethyl-4,8,12-trioxo-3,6,9-triazatricyclo[7.3.1.1^{3,11}]tetradec-6-yl)methyl)indan-2-yl)benzo[b]thiophene-5-carboxamide (**11c**)

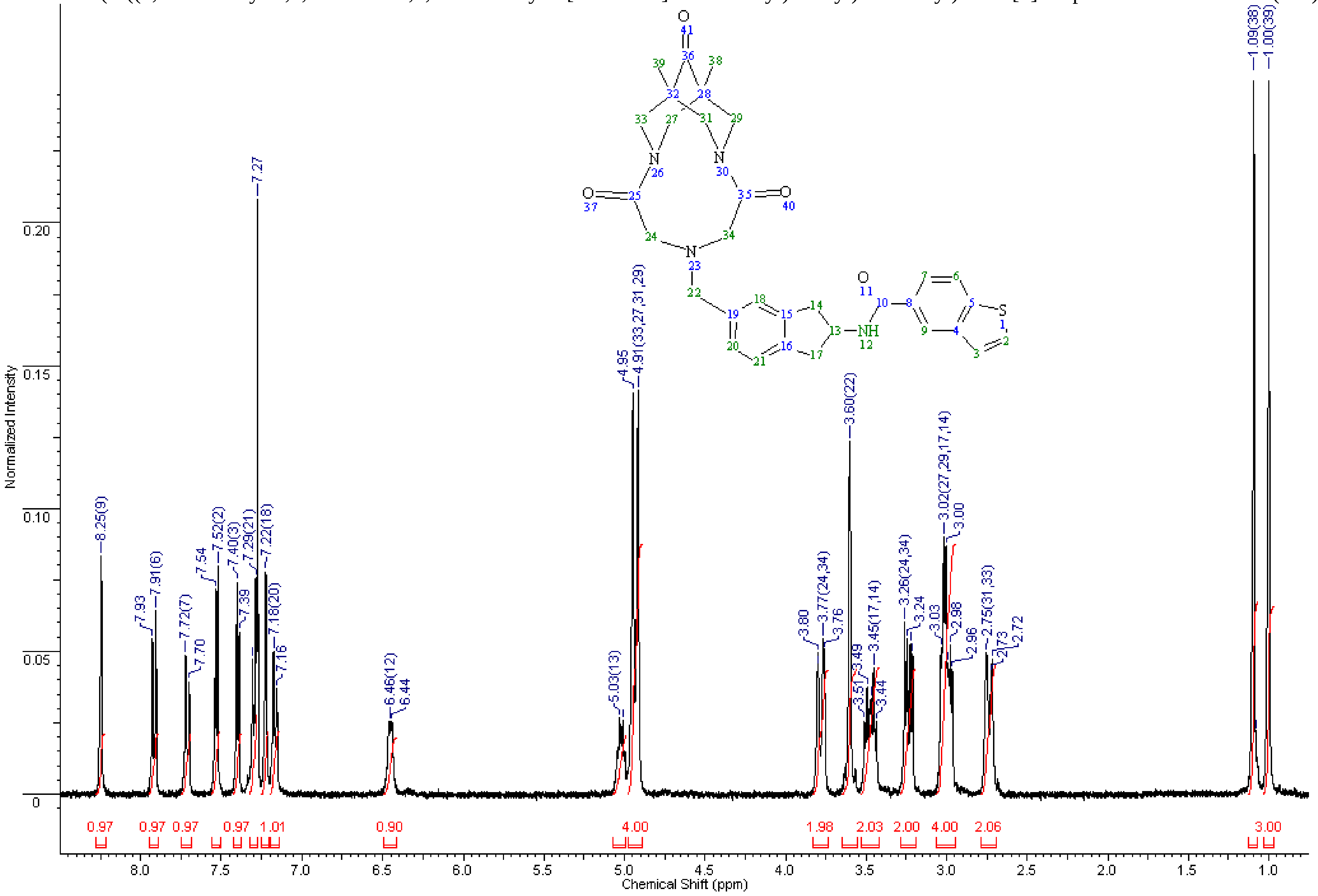

^{13}C NMR N-(5-((1,11-dimethyl-4,8,12-trioxo-3,6,9-triazatricyclo[7.3.1.1^{3,11}]tetradec-6-yl)methyl)indan-2-yl)benzo[b]thiophene-5-carboxamide (**11c**)

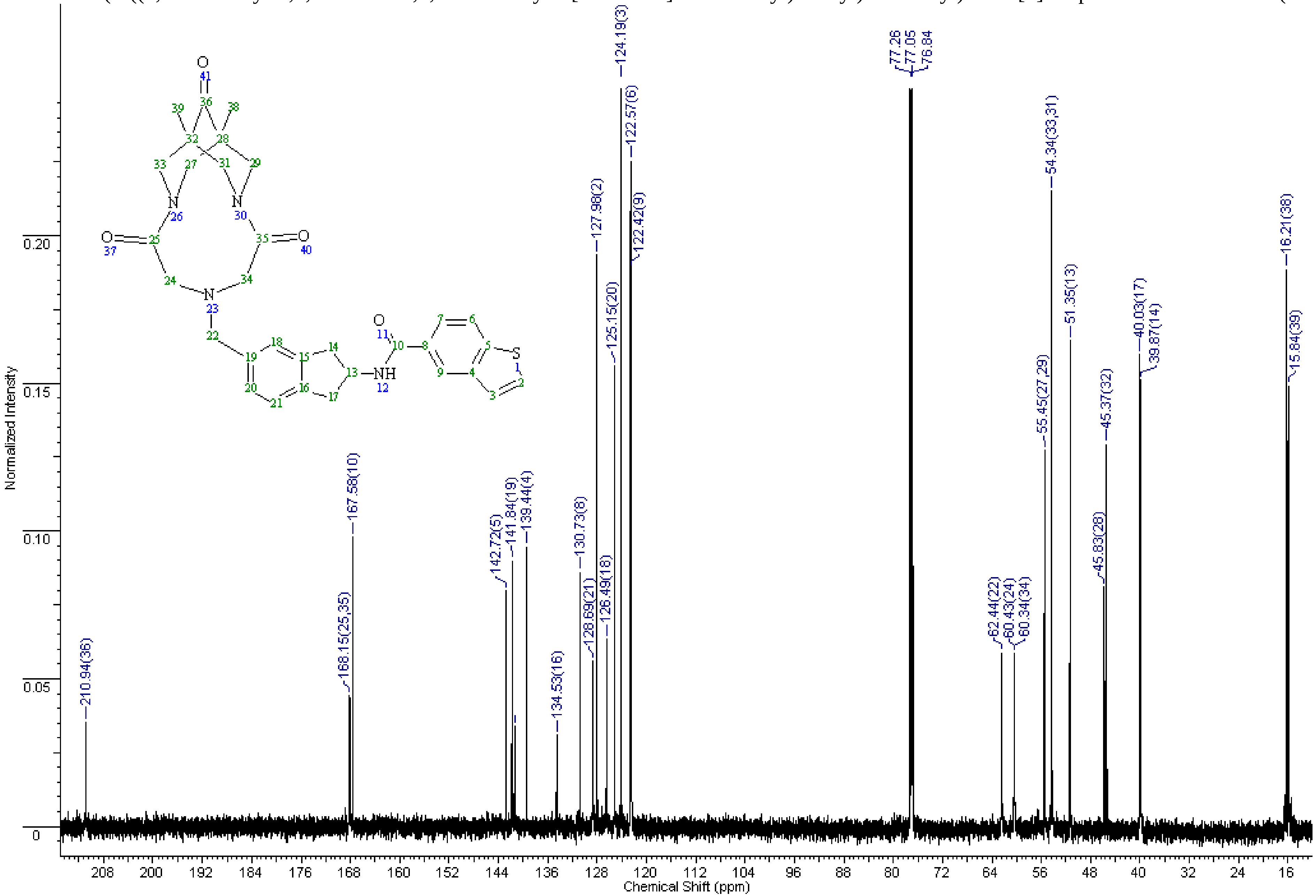

Supplement: Supplementary file 1 [file ijms-24-10293-s001.zip › ijms-2399841-supplementary.pdf]
